# Effective treatment of intractable diseases using nanoparticles to interfere with vascular supply and angiogenic process

**DOI:** 10.1186/s40001-022-00833-6

**Published:** 2022-11-04

**Authors:** Ahmad Hoseinzadeh, Hamed Ghoddusi Johari, Mohammad Hossein Anbardar, Lobat Tayebi, Ehsan Vafa, Milad Abbasi, Ahmad Vaez, Ali Golchin, Ali Mohammad Amani, Ali Jangjou

**Affiliations:** 1 Thoracic and Vascular Surgery Research Center, Shiraz University of Medical Sciences, Shiraz, Iran; 2 Department of Surgery, School of Medicine, Namazi Teaching Hospital, Shiraz University of Medical Sciences, Shiraz, Iran; 3 Department of Pathology, Shiraz University of Medical Sciences, Shiraz, Iran; 4 Marquette University School of Dentistry, Milwaukee, WI 53233 USA; 5 Department of Medical Nanotechnology, School of Advanced Medical Sciences and Technologies, Shiraz University of Medical Sciences, Shiraz, Iran; 6 Department of Tissue Engineering and Applied Cell Sciences, School of Advanced Medical Sciences and Technologies, Shiraz University of Medical Sciences, Shiraz, Iran; 7 Solid Tumor Research Center, Cellular and Molecular Medicine Institute, Urmia University of Medical Sciences, Urmia, Iran; 8 Department of Clinical Biochemistry and Applied Cell Sciences, School of Medicine, Urmia University of Medical Sciences, Urmia, Iran; 9 Department of Emergency Medicine, School of Medicine, Namazi Teaching Hospital, Shiraz University of Medical Sciences, Shiraz, Iran

**Keywords:** Vascularization, Angiogenesis, Anti-angiogenic therapy, Nanoparticles, Therapeutic applications

## Abstract

Angiogenesis is a vital biological process involving blood vessels forming from pre-existing vascular systems. This process contributes to various physiological activities, including embryonic development, hair growth, ovulation, menstruation, and the repair and regeneration of damaged tissue. On the other hand, it is essential in treating a wide range of pathological diseases, such as cardiovascular and ischemic diseases, rheumatoid arthritis, malignancies, ophthalmic and retinal diseases, and other chronic conditions. These diseases and disorders are frequently treated by regulating angiogenesis by utilizing a variety of pro-angiogenic or anti-angiogenic agents or molecules by stimulating or suppressing this complicated process, respectively. Nevertheless, many traditional angiogenic therapy techniques suffer from a lack of ability to achieve the intended therapeutic impact because of various constraints. These disadvantages include limited bioavailability, drug resistance, fast elimination, increased price, nonspecificity, and adverse effects. As a result, it is an excellent time for developing various pro- and anti-angiogenic substances that might circumvent the abovementioned restrictions, followed by their efficient use in treating disorders associated with angiogenesis. In recent years, significant progress has been made in different fields of medicine and biology, including therapeutic angiogenesis. Around the world, a multitude of research groups investigated several inorganic or organic nanoparticles (NPs) that had the potential to effectively modify the angiogenesis processes by either enhancing or suppressing the process. Many studies into the processes behind NP-mediated angiogenesis are well described. In this article, we also cover the application of NPs to encourage tissue vascularization as well as their angiogenic and anti-angiogenic effects in the treatment of several disorders, including bone regeneration, peripheral vascular disease, diabetic retinopathy, ischemic stroke, rheumatoid arthritis, post-ischemic cardiovascular injury, age-related macular degeneration, diabetic retinopathy, gene delivery-based angiogenic therapy, protein delivery-based angiogenic therapy, stem cell angiogenic therapy, and diabetic retinopathy, cancer that may benefit from the behavior of the nanostructures in the vascular system throughout the body. In addition, the accompanying difficulties and potential future applications of NPs in treating angiogenesis-related diseases and antiangiogenic therapies are discussed.

## Introduction

To maintain cell viability under in vivo circumstances, any live mammalian tissue requires oxygen and other nutrients. As a result, blood vessels are critically important in preserving life. Endothelial cells are the most abundant cells in tiny blood vessels, but smooth muscle cells and pericytes surround larger blood vessels walled by endothelial cells [330]. Mainly throughout the human body, the development of new blood vessels (neovascularization) is accomplished by two separate biological mechanisms. The first is called vasculogenesis, while the second is called angiogenesis. Vasculogenesis is creating new and fresh blood vessels from scratch using endothelial cells derived from progenitor cells (such as angioblasts), which are capable of self-assembling into lumens and creating primitive blood vessels as a result of the differentiation of these cells [276, 320].

On the other hand, angiogenesis is the development of new blood vessels due to the branching of blood vessels from the existing vasculature. Angiogenesis is characterized by a sequence of cellular and molecular mechanisms that can be categorized into several stages [184]. This includes the activation of endothelial cells in reaction to pro-angiogenic growth, the degradation of capillary walls through the activity of proteinase enzymes within the extracellular matrix, branch point formation inside the vessel walls, and the migration of endothelial cells into the extracellular matrix afterward towards the starting point of the angiogenic stimulation [207]. Then, the new tubules are connected together to construct a vascular system, which is referred to as an anastomosis in medical terminology [314]. Angiogenesis plays an essential role in embryogenesis and the maintenance of normal homeostasis [9]. Angiogenesis is thought to be dysregulated in a variety of clinical diseases. Even though angiogenesis is inactive or actionless during maturity, it becomes biologically functional in healthy environments like the placenta during pregnancy and the cycling ovary [209]. Moreover, angiogenesis is a common occurrence that happens due to the activation of endothelial cells in response to particular triggers (e.g., hypoxic conditions) that occur throughout the wound healing cycle to expedite tissue regeneration [113]. A different narrative exists concerning undesirable angiogenesis in many illnesses and conditions, namely, how an imbalance between angiogenic inhibitors and stimulators activates the angiogenesis on/off switch [306]. For example, the angiogenesis mechanism is initiated in the presence of cancer or certain inflammatory diseases [3]. In contrast, inadequate angiogenesis is identified in numerous clinical states like ischemic heart tissue, wherein regeneration and repair are impeded due to EC malfunction and vascular regression or deformity [222].

Nowadays, tissue-engineered approaches to improving vascularization can be classified into three types of procedures; the first category includes the vascular growth factors loading into the tissues [274]. Although vascular growth factors have short half-lives and are unstable, there may be challenges in clinical implementation due to these characteristics. The second kind entails in vivo vascularization achieved via co-culturing using endothelial cells in the laboratory [193]. Cell activity and usage patterns, on the other hand, are inferior. The absence of established criteria for the growth and transplanting of seed cells adds to the difficulty of clinical translational healthcare research and development. The third kind makes advantage of micro-engineering technologies enabling the implantation of vascularized mesh; nevertheless, there will still be specific issues to be resolved, including discrepancies in the structural incorporation of blood vessels when microsurgical procedures are employed [254]. As a result, improvements in the precision of vascular system regeneration as well as the interaction of the meshes into tissue are required. Biomaterial scaffolding is widely regarded as a critical element in tissue engineering and regenerative medicine [10, 77, 78]. They contribute to forming the fundamental framework of the tissue structures, which impacts the biological behavior of cells and the availability or effectiveness of growth factors. The ability to adequately use the capabilities of biomaterials as well as the creation of well-designed scaffolding frameworks are crucial for facilitating effective angiogenesis and also subsequent tissue remodeling and regeneration throughout the body.

Nanoparticles (NPs) are substances that have nanometer-range dimensions in at least one dimension. Because of their specific physiochemical properties, as well as their quantum size influences, nanostructures have shown promising possible applications in the areas of tissue regeneration, timely detection, as well as diagnostics [253, 263]. These salient features are owing to the advent of nanotechnology that are updated with extensive studies, from the introduction of unique materials to novel approaches [166, 181, 225, 225–227, 227]. The role of nanostructures in promoting angiogenesis during tissue regeneration is being more widely recognized in the scientific community [133, 171]. Using the caveolae and clathrin receptors, nanomaterials might be endocytosed into endothelial cells or immune cells, inducing alterations in cellular behavior that would aid in the stimulation of angiogenesis [307]. Incorporating nanopatterns on the surface of an implanted vascular graft employing nanofilms can reduce thrombogenicity while increasing the adhesion strength of the implant to circulating endothelial progenitor cells [65]. It has been demonstrated that electrospun scaffold substances, nanofibers, as well as other mesoporous nanoscaffold supplies can be used to mimic the blood vessel’s extracellular matrix, which is advantageous for proliferation, adhesion, endothelialisation, and the migration of endothelial cells inside the vessel walls of the circulatory blood system [48, 65, 115]. In addition, NPs can be used as delivery vehicles to increase the responsiveness and target certain proangiogenic factors, which is an important advancement [34, 327]. A strong case for the usefulness of nano-sized delivery methods for therapeutic pharmaceuticals can circumvent the restricted tissue diffusion of medications, secure them while in the bloodstream, and reduce the danger of systemic side effects [313]. In other words, tailored treatment employing nano-scale transporters makes it easier for drug-loaded nanomaterials to reach the intended places inside the body (organs, tissues, and cells) and the release of drug patterns more consistent and predictable [269].

**Fig. 1:**
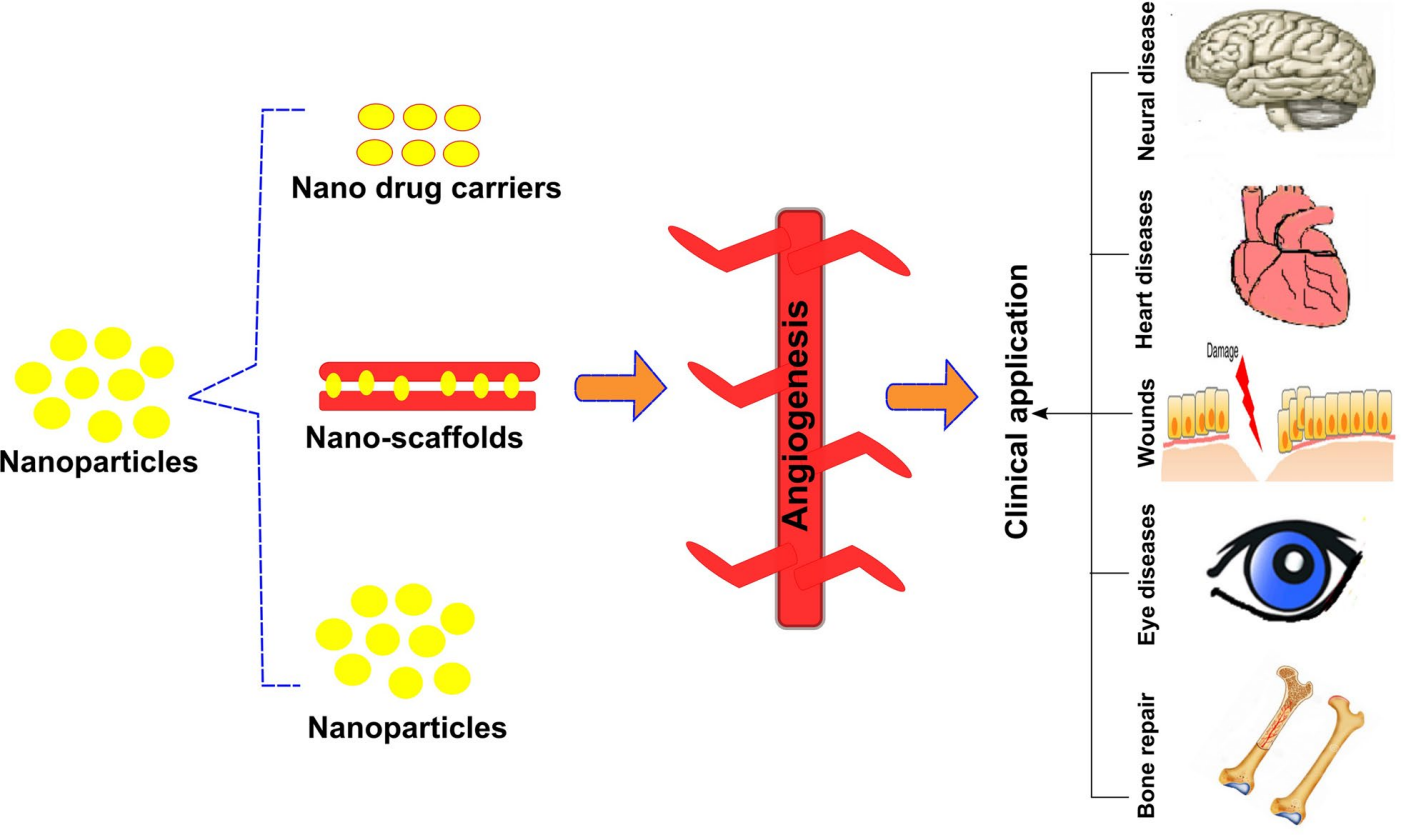
Formulations based on nanomaterials for managing medical diseases characterized by abnormal angiogenesis. Abnormal angiogenesis contributes to the evolution of various disorders, including tumors, diabetic retinopathy, chronic wounds, cardiovascular disease, nerve and bone tissue degeneration, and the wet form of age-related macular degeneration (wet AMD), among others. It is possible to use nanostructures with unique anti-angiogenic or pro-angiogenic properties separately or as a component of biodegradable polymeric scaffolds to manage such illnesses. Various potential nanostructures, some of which have had their surfaces modified with peptides, such as RGD and VEGF, might be used as carrier instruments for targeted drug delivery in various applications. *VEGF* vascular endothelial growth factor, *RGD* arginine–glycine–aspartate

Furthermore, it has been well known that inorganic and organic nanostructures can exhibit antiangiogenic and pro-angiogenic properties based on their nano-sized structural architecture. The use of nanoformulations in pro-angiogenic or anti-angiogenic medicine is a promising technique for managing disorders associated with abnormal vascularization and angiogenesis. Figure 1 depicts a schematic representation of the possible diseases linked with abnormal angiogenesis and the numerous uses of nanostructures for treating such conditions. Numerous newly discovered nanostructures with angiogenic and anti-angiogenic capabilities have been shown to treat various vasculature-related disorders (Table 1).

**Table 1:** Several recently developed nanomaterials with angiogenic and anti-angiogenic properties have been found to cure a wide range of vasculature-related diseases

Disease/ intervention	Nanomaterials	Mechanisms/effects	Refs.
Ischemic Stroke	Hydroxyethyl Starch Functionalized NPs	Sensitively release more smoothened agonists in the acidic environment of ischemic brain tissue, enhance angiogenesis and maintain the integrity of the blood–brain barrier by utilizing the synergistic processes of Pro-His-Ser-Arg-Asn (PHSRN) peptides and smoothened agonist, improve neuroplasticity as well as the rehabilitation of neurological function	[308]
	NIR-driven nanophotosynthesis biosystem	They can be oxygen and absorb carbon dioxide, which allows them to preserve neurons from ischemia and contributes to treating stroke, reducing infarction, enhancing angiogenesis, and facilitating repair of brain tissues	[283]
	siRNA delivery by MRI-visible NPs	Increased angiogenesis, neurogenesis, white matter recovery significantly reduced infarct volume, and functional recovery after ischemic stroke improves therapeutic efficacy of transplanted endothelial progenitor cell	[281, 282, 284]
Peripheral arterial disease	H_2_O_2_-responsive polymer PVAX NPs	rapidly scavenge H_2_O_2_ and release vanillyl alcohol with anti-inflammatory and antioxidant activity, significantly enhancing the expression of angiogenic inducers like platelet endothelial cell adhesion molecule (PECAM)-1 and vascular endothelial growth factor (VEGF) in human umbilical vein endothelial cells (HUVEC), induced revascularization and blood perfusion restoration into ischemic tissues through upregulating angiogenic PECAM-1 and VEGF	[136]
	Indocyanine green-loaded boronated maltodextrin NPs	They exhibited H_2_O_2_-triggered amplification of ultrasound fluorescence, and photoacoustic signals in the ischemic hindlimb muscles, potent anti-inflammatory and proangiogenic activities	[114]
Bone regeneration	Dexamethasone-loaded biphasic calcium phosphate NPs/collagen composite scaffolds	simultaneous promotion of osteogenesis and angiogenesis, microgrooves in the scaffolds were intended to direct the assembly of HUVECs into well-aligned tubular frameworks, which would then promote fast angiogenesis	[46]
	3D scaffold based on poly (L-lactic acid) (PLLA)/Polycaprolactone (PCL) matrix polymer consisting of gold nanoparticles (Au NPs) and gelatin nanofibers	The most significant levels of neo-bone development, osteocyte in lacuna weaved bone creation, and angiogenesis was seen in the defect location	[234]
	K-doped zinc oxide (ZnO) NPs containing porous hydrogels	Significantly induce neovascularization and angiogenesis	[245]
Diabetic retinopathy	Hydrogel comprising dexamethasone/Avastin-loaded Chitosan-N-acetyl-L-cysteine NPs	The optimized formulation could improve diabetic retinopathy, improve concomitant vitreoretinal disorders, improve treatment of posterior eye segment neovascularization,	[258]
	IL-12-loaded poly(lactic-co-glycolic acid) (PLGA) NPs	They showed better inhibitory efficacy against MMP-9 and VEGF-A expression in diabetic retinopathy mouse retina and rat endothelial cells, decreased neovascularization, increased thickness, reduced retinal damage	[321]
Rheumatoid arthritis	Methotrexate-loaded mannose-modified human serum albumin NPs	Angiogenesis in chick embryos was significantly inhibited, arthritic joints were the primary site of the accumulation of NPs, considerable reduction in the serum levels of inflammatory cytokines, as well as joint bone degradation and swelling, showed analgesic effects	[162]
	SeNPs–PEG–RGD@Ru	They promote uptake by HUVECs and trace the biodistribution and internalization, the extensive neovascular network of inflammatory locations is what these NPs aim to target to produce; NO, stimulate the apoptosis of HUVECs, and prevent the formation of new vessels in the localized tissue, NO activates autophagy through the modulation of signaling pathways associated with mTOR and AMPK, the increase of the flux of autophagy, the inhibition of the function of NF-B-p65, and the modulation of the concentrations of inflammatory cytokines	[154]
Critical limb ischemia	Cerium oxide NPs	They exhibited pro-angiogenic activity in a mouse hindlimb ischemia model, mice given 0.6 mg of cerium oxide had a higher rate of limb salvage and blood vessel reperfusion in the hindlimbs, they increased endothelial survival by scavenging ROS, revascularize an ischemic limb by promoting Ref-1/APE1-dependent angiogenesis	[205]
Age-related macular degeneration	Sodium butyrate-loaded NPs coated chitosan	They inhibited the angiogenesis in CAM assay, showed no toxicity to human retinal pigment epithelium cells (ARPE-19 cells), did not interfere in the integrity of the retinal layers of rat’s eyes	[220]
	Resveratrol-loaded PLGA NPs	They displayed in vitro anti-angiogenic properties by inhibiting expression of VEGF	[29]
Stem cell-based intervention	Hybrid quinacrine and gold NPs	They caused apoptosis in vitro, significantly inhibited cellular proliferation, disrupted tumor regression in xenograft mice model and disrupted angiogenesis in vivo, inhibited crucial angiogenic markers VEGF, Ang-2, and Ang-1, depleted MMP-2 in H-357-PEMT cells in a p21 and p53-dependent manner, increased the generation of NO and ROS, educed the mitochondrial membrane potential	[238]
	Poly(N‑isopropylacrylamide) (PNIPAM) NPs loaded with collagen	After 14 days in the osteogenic culture media, endothelial differentiation and capillary-like tube formation were verified, Expressions of osteogenic markers such as runt-related transcription factor 2 (RUNX2), osteocalcin (OCN), and collagen type I (Col I) were confirmed, as were expressions of angiogenic markers like kinase insert domain receptor (KDR), von Willebrand factor (vWF), and platelet–endothelial cell adhesion molecule-1 (CD31)	[2]
Cancer	RGD-Glycol chitosan NPs modified with 5β-cholanic acid	These NPs were capable of reducing HUVEC adherence to a βig-h3 protein-coated surface, which suggests that the RGD peptide included in the NPs has an antiangiogenic effect; as a formulation of an antiangiogenic peptide medication, the NPs effectively reduced bFGF-induced angiogenesis and lowered hemoglobin levels in Matrigel plug	[128]
	Nanoceria	Nanoceria strongly decreased the generation of ROS in A2780 cells, and it slowed the invasion and migration of SKOV3 cells that were driven by growth factors (HB-EGF, VEGF165, SDF1, and HGF). However, it did not influence the cell proliferation, treatment with nanoceria was capable of preventing VEGF165-induced proliferation, in addition to the development of capillary tubes and the activation of MMP2 and VEGFR2 in HUVEC, attenuation of angiogenesis was found, which was verified by lower CD31 staining and selective death of vascular endothelial cells. This attenuation occurred concurrently with the reduction in tumor mass	[76]

In this paper, we discuss the application of NPs to encourage tissue vascularization as well as their angiogenic, proangiogenic, and anti-angiogenic effects in the treatment of several disorders, including peripheral vascular disease, diabetic retinopathy, bone regeneration, rheumatoid arthritis, ischemic stroke, post-ischemic cardiovascular injury, diabetic retinopathy, age-related macular degeneration, protein delivery-based angiogenic therapy, gene delivery-based angiogenic therapy, stem cell angiogenic therapy, diabetic retinopathy, anti-angiogenic therapy, and cancer that may benefit from the behavior of the nanostructures in the vascular system throughout the body. In addition, the challenges with treating angiogenesis-related disorders and the possible future uses of nanoparticles in antiangiogenic treatments are highlighted.

## The importance of angiogenesis and anti-angiogenesis

As a role in several physiological processes, angiogenesis is critical throughout one's life. These courses include hair growth, wound healing, embryonic and ovulation development, tissue regeneration, organ development, and the menstrual cycle [13, 70, 189]. Given that angiogenesis is strictly regulated by the interaction of inhibitors and growth factors, any imbalance in their levels can result in catastrophic illnesses, such as ischemic and cardiovascular disorders, cancer, ocular diseases, delayed wound healing, and other conditions[94, 137, 316]. The abnormal angiogenesis mechanism that occurs in diabetic retinopathy and cancer provides nourishment to the damaged tissue, and as a result, these disease states may be treated using biomolecules inhibiting the angiogenesis [132, 262]. On the other hand, pro-angiogenic growth factors are utilized to treat ischemic or cardiovascular disorders and prolonged wound healing, since these clinical states are connected with inadequate blood vessel development, which results in poor blood supply and tissue loss. It is essential to identify diverse anti-angiogenic and pro-angiogenic materials or molecules that might be used to treat various disease-related to angiogenesis, given the vital role that angiogenesis plays in both pathological and physiological processes.

The vasculature systems that supply tumors are very abnormal in comparison to those that compose the normal vascular system. These abnormal blood vessels are highly permeable, tortuous, and diverse in their morphological structure and perfusion effectiveness [86]. These characteristics are what constitute what is now known as "aberrant angiogenesis," which is what distinguishes the environment of the tumor[279, 285]. Ineffective medication delivery to cancerous cells is one of the challenges facing oncology researchers today, and it's one of the reasons why cancer therapy is often unsuccessful. Fenestration of the tumor vascular system is caused by a lack of adequate interconnections of the endothelial cells (ECs) throughout the tumor. This is a serious roadblock to the delivery and equal distribution of chemotherapeutics to the tumor tissue [183]. When an anti-angiogenic medicine is combined with an anti-tumor therapy during the optimal time window, the balance between pro- and anti-angiogenic factors is restored [183]. These results in the blood vessels' normalization, which enables the chemotherapeutic agent to enter the targeted tumor site. These effects are restricted in space and time and vary depending on the cancer being treated, especially when dealing with tumors that do not have a significant blood supply. The choice of a precise and individualized therapy targeted at the normalization of the blood vessels requires the detection and prediction of the architectural features of the microvessels [147]. Another feature of tumor vasculature is the absence of pericytes, which results in the vessel wall being thinner, thus altering the permeability both within a single tumor and throughout various tumors [332]. As a result of aberrant vascularity, tumors often develop a resistance to chemotherapeutics. The delivery of chemotherapeutics as a treatment method for various cancers is assisted by the inclusion of molecules having anti-angiogenic activities [235]. These molecules aim to inhibit tumor development by lowering the number of blood vessels that develop within a tumor.

## Ischemia stroke

In a person's lifetime, one in every four will suffer from a stroke, which is the primary cause of death and disabilities in adults worldwide [62]. In the medical community, stroke is described as a neurological deficiency caused by an acute focused central nervous system lesion caused by an artery [135]. Ischemic stroke is now treated by intravenous infusion of human endovascular thrombectomy and plasminogen activator in the human tissue (intravenous thrombolysis), both considered emergency therapies. Intravenous thrombolysis (IVT) is a procedure that uses enzymes to dissolve the thrombus that is provoking the stroke [23]. This therapy is administered in the first 4.5 h following the beginning of the stroke [40], although endovascular thrombectomy (EVT) has a high rate of success in patients who have had a stroke due to a significant artery blockage and can be administered in chosen individuals up to 24 h after the onset of the stroke, albeit its effectiveness is highly reliant on the timing of the stroke [203]. It is conducted in an angiography room, in which a catheter is put into an artery by an experienced physician and tracked using X-ray image analysis until it reaches and removes the blood clot. The procedure takes about an hour. Many stroke survivors experience restricted functional recovery due to inadequate restructuring and repair processes inside the lesion region, which is unfortunately common. Recent years have seen the development of neuroprotective techniques that address the cascade of molecular and cellular processes that contribute to ischemia damages as well as initiatives to stimulate post-ischemic rehabilitation, albeit practical translation has not yet been achieved.

Since then, sophisticated therapeutic and diagnostic techniques based on cell-based treatments, novel pharmacological substances, and appropriate biomaterials have been submitted for consideration [156, 182]. One technique that has received significant interest is nanostructures' employment for diagnosing and treating in medical applications [38, 196, 204]. The goal here is to extend the duration of medicines inside the bloodstream and thus improve their penetration across the blood–brain barrier (BBB) to enter the ischemic region. The majority of these techniques have only been tested in pre-clinical animal studies and, as a result, have not yet been tested in humans. However, although recent studies have focused on nanomaterials in the context of stroke, there has been little discussion on the use of nanostructures to address the required specifications associated with the various stages of ischemic stroke [35, 61].

### NPs in the stroke diagnosis

NPs are essential for molecular-based brain image analysis, because they can uncover biochemical mechanisms that could be used as therapeutic and diagnostic strategies for stroke [27]. To achieve high-resolution imaging of the cerebral vasculature, which is the main objective in stroke investigations, brain imaging based on nanotechnology can provide a far more precise image of the extent of the ischemic lesion [64].

Computed tomography (CT) and magnetic resonance imaging (MRI) are the primary imaging techniques used in stroke diagnosis [215]. Several applications for these image processing instruments include the detection of stroke in its initial phases, evaluating the most pertinent pathophysiological features of stroke, like BBB interruptions, and the classification of patient populations who will advantage from tissue plasminogen activator recanalization therapy. The utilization of nanostructures in ischemic stroke research has included the adoption of iron oxide NPs as a contrast agent for timely diagnosis of neuroinflammation through MRI [249] and the use of α_v_β_3_integrin-targeted NPs to track pro-angiogenic responses throughout ischemic stroke region, especially in diabetic experimental animals [17]. Aside from that, clinical trials have shown that ultra-small superparamagnetic iron oxide NPs can improve MRI contrast, which allows for safe surveillance of post-stroke inflammatory response by tracking macrophage migration into the ischemic brain [233]. Even though CT scanning has a lower sensitivity/specificity than MRI, it is employed for molecular imaging in stroke. As a result, gold nanomaterials coupled with fibrin peptides have demonstrated the capacity to increase picture detail when showing brain vascular thrombus using CT [123, 124, 127].

### Inorganic NPs as ROS Scavengers

Investigations have demonstrated that metal NPs can act as scavengers of ROS. Ischemic stroke is responsible for the production of ROS, which include hydrogen peroxide, hydroxyl radical, and superoxide anion. Ischemic stroke causes brain damage by a oxidative tissue damage mechanism, and ROS plays a role in this process [148].

There is evidence to suggest that ceria NPs have the ability to scavenge free radicals through reversibly interacting with oxygen and generating oxidized Ce^4+^ species from reduced Ce^3+^ species [122]. First, Kim et al. found that ceria NPs inhibited ROS formation and stopped apoptosis in cells, which protected the brain from ischemia injury. The BBB integrity is the obstacle that prevents ceria NPs from depositing in the brain. Ceria NPs (E-A/P-CeO_2_) modified using Angiopep-2 (ANG) as well as poly(ethylene glycol) were designed by Bao and colleagues. These NPs were successful in crossing the BBB by the process of receptor-mediated transcytosis. The results of an in vitro transmigration study showed that E-A/P-CeO_2_ had a greater ability to pass through the brain capillary endothelial cells (BCECs) than P-CeO_2_ [21]. The capacity of E-A/P-CeO_2_ to penetrate the BBB was also demonstrated by testing it on healthy rats by calculating the ratio of ceria NPs present throughout brain tissue and injecting them. Last but not least, the findings demonstrated that E-A/P-CeO_2_ has the capacity to scavenge ROS and reduce the number of infarcts in rats. According to the findings, ceria NPs have the potential to be useful in the treatment of I/R damage.

Platinum NPs are considered to be innovative ROS scavengers due to the fact that their ability to enhance catalytic properties to quench ROS can be attributed to their high electron density and large surface area [289]. In a mouse model of transient middle cerebral artery occlusion (tMCAO), Takamiya et al. explored whether or not platinum NPs with a size range of 2–3 nm have a neuroprotective impact against ischemia and reperfusion injuries [260]. The findings demonstrated that treatment with platinum NPs reduced the extent of mouse infarcts as well as improved the formation of superoxide in the cerebral cortex through a decrease in the levels of hydroethidine. The same research team released another study in which they investigated whether platinum NPs may reduce ischemia damage caused by tissue plasminogen activator (tPA). This was done, since therapy with tPA may upregulate the MMP-9 expression. It does this through a protein called low-density lipoprotein receptor-related protein [259]. This makes the cerebral infarction severe. They discovered that the presence of platinum NPs reduced the activity of MMP-9 and improved the state of the damaged neurovascular unit (NVU) caused by tMCAO. These results provided additional support for the hypothesis that platinum NPs might be used with tPA reperfusion therapy to treat individuals suffering from ischemic stroke.

### NPs in the treatment of subacute phase of stroke

Described as the ischemic stroke’s subacute phase, this phase outlines the regeneration activities that occur approximately 1 week after the initiation of the stroke. Angiogenesis is a critical component of the restorative processes during this period, since it contributes to the physiological, instead of pathological, rise in permeability in the BBB and the restoration of functional abilities following a stroke. The endothelial progenitor cell’s mobilization to the infarct area begins during the acute phase in reaction to the pro-inflammatory secretion of growth factors and chemokines, including angiopoietin-2 (Ang-2) and vascular endothelial growth factor (VEGF), released by hypoxic cells [28]. The rapid activation of angiogenesis and overexpression of VEGF are associated with an improved permeability of the BBB, which may raise the risk of heart attack and stroke. Increased capillary thickness with more considerable vessel localization in the penumbra and improved collateral circulation has been related to overexpression of Ang/Tie-2 and VEGF in later stages of the disease [237]. A change in tight junction structure occurs in the late subacute phase, with the assistance of activated protein C and sphingosine-1-phosphate, which significantly reduces BBB permeability in newly formed vessels that have matured throughout the subacute stage but are still in the developmental stage. Currently, rehabilitation on both a cognitive and physical basis is used to treat patients in the chronic phase [176]. This method's beneficial result is that the neurovascular unit has been repaired, and neurogenesis has been encouraged in the neurogenic niches in the lateral ventricle’s subventricular zone and the subgranular zone of the hippocampal dentate gyrus. Astrocytes release several substances that participate in the processes during the chronic phase, including VEGF and primary fibroblast growth factor (bFGF). Specifically, in neural stem cells (NSC), bFGF increases the VEGF receptor Flk-1 expression and VEGF as a neurotrophic factor, promoting their migration and proliferation [301, 325, 328].

As nanomaterials provide a more extended temporal window for therapeutic efficacy, novel techniques for vascular protection and angiogenesis during subacute stroke are being developed to address these issues. It is polymeric NPs that are most frequently used to deliver angiogenic agents in the subacute phase of acute ischemic strokes [27, 109, 298]. Various polymeric materials have distinct physicochemical qualities that rely on the characteristics of the basic building block that they are constructed from. Many polymeric NPs may be functionalized and can even be pharmaceutically conjugated. NPs have been demonstrated to be beneficial in managing angiogenesis throughout the subacute stage [57, 106]. When bFGF and stromal cell-derived factor 1 (SDF-1) was delivered in the exact location by a hydrogel comprising polymeric NPs, angiogenesis and neurogenesis were greatly enhanced, while the infarct volume was increased substantially decreased [106]. Following permanent middle cerebral artery blockage, the releasing of SDF-1 inside the ischemic area triggered via the dual-ionic copolymer poly(urethane amino sulfamethazine) (PUASM) responsive to pH changes also resulted in increased angiogenesis in the ischemic boundary zone [123, 124, 127].

Further investigation revealed that hypoxia-inducible factor 1α (HIF-1α) was filled with polymeric cationic nanomaterials, which were surface layered with the RGD (arginyl glycyl aspartic acid) peptide, performed a practical function in vascular rehabilitation as demonstrated by visual evaluation of the zebrafish. This means allowing for simple experimental intervention and provides an attractive method for understanding cerebral ischemia as well as angiogenesis. Three days following treatment with this formulation, the therapeutic efficacy of the formulation was investigated in an ischemic stroke rat model, which revealed that the infarct amount had significantly decreased [57].

While the subject of nanomedicine is extremely promising in the context of ischemic stroke, nanostructures have significant limitations that prevent them from being used in healthcare situations. The hazards related to nanotoxicity, which can result in neuroinflammation, death of cells, and disturbance of the BBB, highlight the necessity for better methodologies for assessing neurotoxicity before NPs are approved for human consumption. Furthermore, the requirement for large-scale clinical studies to establish the safety of NPs in ischemic stroke patients restricts their ability to be translated into the clinic promptly.

### NPs for promoting revascularization in post-ischemic cardiovascular injury

Restoring blood flow to the coronary arteries in a timely manner is presently the most efficient treatment for treating acute myocardial infarction [219]. The current pandemic of heart failure, on the other hand, implies that it is not enough to simply recanalize occluded arteries quickly after the beginning of myocardial infarction [168]. Ischemia–reperfusion injury occurs when blood flow is restored to ischemic myocardium. This results in the death of cells and an increase in the size of the infarct region. The therapeutic use of these medications has not been successful as of yet, despite the fact that hundreds of compounds have been tested on animals to determine whether or not they have the capacity to minimize ischemia–reperfusion injury [116, 175]. In acute myocardial infarction, the opening of the mitochondrial permeability transition pore and inflammatory responses both contribute to the progression of cardiac ischemia–reperfusion injury, which in turn hinders the therapeutic benefits of primary reperfusion treatment. Gentaro Ikeda and colleagues investigated the potential therapeutic benefits of nanoparticle-mediated medications that concurrently influence the mitochondrial permeability transition pore and inflammatory processes in the context of ischemia–reperfusion damage [101]. Enhanced cardioprotection following myocardial ischemia–reperfusion injury was observed when concurrent targeting of CypD-knockout-mediated mitochondrial permeability transition pore opening and inflammatory processes was performed. To prevent the heart from ischemia–reperfusion injury in patients who have had an acute myocardial infarction and have undergone revascularization, PLGA nanoparticle-mediated delivery of cyclosporine A and pitavastatin towards the heart after ischemia–reperfusion injury can be a clinically viable and efficient strategy. Recently, to treat myocardial ischemia, melatonin–biosmart NPs featuring microenvironment targeting and self-adaptive capacity (MTSNP) have been designed by mimicking the function and structure of mitochondria [151]. Melatonin can help preserve the structural integrity of mitochondrial membranes and also possesses antioxidant properties. The twin PLGA shells that make up MTSNP are analogous to the two-layered membranes that are seen in mitochondria. The melatonin-loaded cores are designed to replicate the cell-protective mitochondria mechanism by functioning similarly to the matrix of the mitochondria. The role of the circular DNA that is found in the space between the two PLGA shells is comparable to that of the mitochondrial DNA. Following injection of MTSNP into the myocardium, melatonin was promptly released from the MTSNP, where it inhibited the release of cytochrome c by binding to the melatonin receptor I on the mitochondrial membrane. This successfully prevented apoptosis in cardiomyocytes during the acute stage of ischemia. The circular DNA would thus be capable of detecting hypoxia and creating VEGF, which would facilitate revascularization. In the mouse model of myocardial ischemia, therapy with MTSNP was shown to reduce the size of the infarct and promote revascularization. Wen Ai and colleagues investigated the potential curative benefits of bilirubin nanoparticles on mice with cardiac ischemia reperfusion damage [6]. After myocardial ischemia and reperfusion, they demonstrated that bilirubin NPs may precisely target the damaged location in the heart. After ischemia reperfusion, the size of the infarct was dramatically decreased as a result of the targeting and ROS-scavenging capabilities of bilirubin NPs. Furthermore, heart function was evaluated using echocardiography and pressure–volume loops, which demonstrated that administration of bilirubin NPs considerably reduced the severity of ischemia reperfusion-induced cardiac dysfunction. It is quite likely that the antioxidant activity of bilirubin was the underlying mechanism that allowed bilirubin NPs to exert their protective benefits. The administration of bilirubin nanoparticles led to the prevention of ROS-induced apoptosis and inflammation in cardiomyocytes, in addition to timely revascularization. Yasin Oduk and colleagues investigated to know if it was possible to accomplish prolonged exposure to a low dose of VEGF by encapsulating VEGF in polylactic coglycolic acid NPs [194]. They also wanted to know if treatment with VEGF-containing NPs continued to improve cardiac function and was designed to protect against left ventricular remodeling in the hearts of mice that had been given an experimentally stimulated myocardial infarction. The encapsulation effectiveness was 53.5 ± 1.7% (107.1 ± 3.3 ng VEGF/mg NPs), and polylactic coglycolic acid NPs featuring an average diameter of 113 nm were created through double emulsion and packed with VEGF. VEGF NPs were capable of releasing VEGF continuously for a period of 31 days when grown in culture. When used in a murine model of myocardial infarction, administration of VEGF NPs was related to reductions in infarct size, significantly greater vascular density in the peri-infarct region, and left ventricular contractile function improvements 4 weeks after treatment. Therefore, the findings of their study prove in concept that the angiogenic and therapeutic potential of VEGF can be increased by the use of NP-mediated administration in the therapeutic intervention of ischemic heart disease.

When it comes to the distribution of drugs, a good number of nanocarriers depend on the EPR effect to extravasate from the circulation and arrive at the chosen target location. The EPR effect is brought on by aberrant vasculature or vasculature that is particularly permeable at the sites of damage or inflammation. The EPR effect that develops after a myocardial infarction is not brought about by the same process [299]. During a myocardial infarction, a significant number of cardiac cells, particularly cardiac endothelial cells, are lost in a short amount of time. As a result of the release of cytokines, there is a rise in the vascular permeability or inflammatory processes of the immediate area [169]. In spite of this, the infarcted myocardium has a rather weak EPR impact when it comes to nanomedicines. Following an ischemia–reperfusion injury in mice, a research team evaluated the biodistribution of fluorescently tagged PEG-modified nanostructures ranging in size from 20 to 2000 nm using a range of particle sizes [158]. After allowing the NPs to circulate for 30 min, the vascular system of the mouse was perfused to eliminate any unbound NPs from the arteries, and then samples of the tissue were taken. Therefore, approximately 0.029 percent of the 20 nm NPs that were introduced into the normal myocardium were maintained. This rose by roughly 5.5 times after the ischemia–reperfusion injury, which demonstrates that an EPR effect is actually present in the left ventricle that has been infarcted. In spite of the fact that this increment was statistically meaningful, it reflects such a modest level of NP delivery (below 0.2%) that it is extremely doubtful whether it is clinically important. To put this into perspective, the liver and spleen collectively stored more than fifty percent of the NPs that were administered. Using NPs with a size of 500 nm resulted in the largest overall absorption by the heart following myocardial infarction (0.27%); nevertheless, confocal imaging showed that the majority of these nanomaterials were trapped within tiny capillaries instead of in the myocardium itself.

Researchers led by Bo-fang Zhang investigated the possibility that magnetized endothelial progenitor cells (EPCs), when directed by an externally applied magnetic field, might enhance the aggregation of EPCs in an ischemic location, hence increasing the therapeutic effectiveness of the treatment. To create magnetized EPCs, the EPCs from male rats were first separated, and then they were tagged with silica-coated magnetic iron oxide NPs [323]. After that, the markers of revascularization, migration, proliferation, and cytophenotypic characteristics of magnetized EPCs were investigated. Then, at 7 days after myocardial infarction, the magnetized EPCs (1 × 10^6^) were delivered into a female rat model of the condition through the tail vein, either with or without the use of an external magnet placed above the infarct region. The transplantation of magnetized EPCs directed by an external magnet resulted in a considerable improvement in cardiac function, a reduction in infarction size, and then a reduction in myocardial apoptosis in rats with myocardial infarction. In addition to this, improved aggregations of magnetized EPCs were seen in the infarcted border zone of rats that had undergone external magnet-guided transplantation. This was accompanied by a substantially enhanced microvessel density and an upregulated expression of proangiogenic factors in comparison to rats that had not undergone external magnet-guided transplantation. This improved aggregation of EPCs in the infarcted border zone was connected to the magnetic field-guided transplantation of magnetized EPCs, which led to an improvement in the therapeutic effectiveness of myocardial infarction.

## Bone regeneration

Through the delivery of oxygen and nutrients and interactions between osteocytes and endothelial cells, the vascular structure that develops in a bone defect stimulates the differentiation, migration, and bone formation of osteoprogenitor cells [273, 325, 328]. Bone development is impaired and delayed when vascularization is restricted or disrupted. The absence of angiogenesis in the defective region is the primary reason for the absence of osteogenesis following in vivo implantation. Several angiogenic factors, including HIF-1α and VEGF, have significantly increased osteogenesis and osteoblast development [100, 229, 271]. As a result, efficient vascularization is required to promote functional restoration and repair bone defects.

There have been several reports of potential nanostructures that are beneficial in enhancing bone tissue regeneration [161]. According to a study, synthetic injectable gel chitin–CaSO_4_–nano-fibrin system demonstrated accelerated osteo-regeneration through increased angiogenesis [134]. Furthermore, the β CaSiO_3_/PDLGA composite (PDLGA: Poly(D, L-lactide-co-glycolide) copolymer formulations) has been shown to promote the activation and phosphorylation of endothelial NO synthase (eNOS) and Akt in human umbilical vein endothelial cells (HUVECs), resulting in an increase in the production and release of VEGF and nitric oxide (NO). Another investigation in which a CaSiO3/PDLGA composite was used to evaluate bone regeneration in a rabbit model with a femur defect found increased osteogenesis and angiogenesis [280, 288]. By decreasing NO production and phosphorylation of eNOS, nano-hydroxyapatite has been shown to modulate the PI3K/Akt pathway, which is responsible for suppressing tube development and migration in HUVECs [246]. In addition, it has been observed that calcium phosphate, in combination with electro spun poly (lactic acid) can stimulate VEGF production by endothelial cells in the same way. When injected subcutaneously into mice, it has also been shown to promote the expression of proangiogenic factors, such as insulin-like growth factor 2 (IGF-2), interleukin 6 (IL-6), VEGF, interleukin 1 beta (IL-1β), interleukin-12 (IL-12p70), granulocyte–macrophage colony-stimulating factor (GM-CSF), and others, therefore, stimulating bone regeneration as well as vascular growth [195]. Another substance that can stimulate bone regeneration and angiogenesis is nanobioactive glass, which has a three-dimensional (3D) channel framework and a larger surface area [240].

Nanostructures can serve as delivery vehicles for various small molecules with pro-angiogenic properties and for transporting proteins, such as adrenomedullin, VEGF, and deferoxamine, among others. For instance, a 3D nanofibrous gelatin (GF) framework incorporating mesoporous silicate nanomaterials (MSNs) has been used to transport both deferoxamine (DFO) and bone morphogenetic protein-2 (BMP2) simultaneously. Because DFO is a hypoxia-mimetic medication, it has the potential to cause the stabilizing of HIF-1α and the consequent induction of angiogenesis. It has also been demonstrated that DFO may dramatically improve osteogenic differentiation induced by BMP2 in human and mouse stem cell models [312]. Ionic elements have been used to modify the engineered vascularized bone tissue scaffolds in bone regeneration. Copper-containing nanostructures were shown to increase the amount of VEGF expression, increasing the proliferation of endothelial cells. When exposed to Cu2^+^-containing solutions during hydrothermal treatment, nano-sized surfaces on Hydroxyapatite frameworks/scaffolds might affect the proliferation of endothelial cells. Furthermore, the nano-sized surfaces over the Hydroxyapatite frameworks were shown to stimulate angiogenesis as well as bone regeneration in the survey respondents [272]. When dexamethasone (DEX) was mixed with a biphasic calcium phosphate nanostructure (BCP NPs) structure, it was shown that the VEGFR2 and VEGF expression was increased which helped to promote bone repair. Micro-grooves facilitated angiogenesis within the frameworks, which controlled the assembling of HUVECs into tubular architectures. Magnetic microspheres packed with genes also have been employed as a potential delivery mechanism [46]. For example, the co-administration of superparamagnetic (nano-Fe_3_O_4_) chitosan and VEGF165 resulted in bone regeneration and angiogenesis induction both in vivo and in vitro [159]. It has also been observed that the gold NPs can stimulate the angiogenesis process during the osteogenesis cycle. In terms of angiogenic activity, the exterior charges of gold NPs and the availability of functional groups were found to differ depending on the kind of functional group. The results of the gene profiling showed that, in comparison to the cells (hMSCs) treated with gold NPs having hydroxyl or amine functional groups, the treated cells using gold NPs having carboxyl group had increased levels of expression of fibroblast growth factor 2 (FGF2) and transforming growth factor-beta (TGF-β), which then turn stimulated cell proliferation rather than osteogenic differentiation [145].

## Peripheral vascular disease (PVD)

Over 8 million people in the United States are affected by peripheral vascular disease (PVD), making it one of the most common vascular disorders in the country. The percentage of the elderly population in the United States (US) affected by PVD ranges from 12 to 20 percent and is known to increase with age [223, 248]. In general, PVD refers to the blockage or constriction of the arteries outside of the heart (non-myocardial arteries). This condition most frequently affects the lower extremities, although it can also affect the vascular system of the kidney and other vascularized organs. The lack of blood flow causes the tissue to be gradually deprived of oxygen and nutrients, which can lead to symptoms like sores, ulcers, claudication, and a change in the skin color of affected limbs. If appropriate therapies are not carried out promptly, a condition known as critical limb ischemia (CLI) can develop, which poses a significant threat of amputation of the affected limb(s) [14, 198, 270]. In both the United States and Europe, it is assumed that the average annual incidence of CLI is between 5 and 10 new cases per 100,000 people, with type-2 diabetes being one of the most important risk factors [174]. Lesions on the skin (such as gangrene or ulcers) and discomfort at rest are two symptoms linked with CLI. Both of these symptoms can drastically lower a patient's quality of life. The CLI has been linked to extremely high mortality and morbidity rates. Researchers have reported that 30% of patients who are not candidates for surgical revascularization will require a severe amputation and that 25% of these patients will die within one year of the beginning of CLI [191].

To date, a great number of methods have been developed to revive blood perfusion in ischemic tissues, which has resulted in the alleviation of rest pain, the healing of ulcers, and the prevention of limb amputation. In instances of localized macrovascular disease, currently available therapeutic interventions like atherectomy, bypass surgery, stent implantation, and angioplasty have the potential to be successful. Unfortunately, these traditional treatments have several drawbacks that limit their effectiveness. First, the intrusive nature of mechanical revascularization procedures frequently makes them inapplicable to a subset of PVD patients who are medically unfit to go through significant surgical procedures. These patients have been diagnosed with PVD [59, 192]. Second, when it comes to bypass procedures, the application of autologous vascular grafts is restricted by the patient's current state of health, while standard artificial grafts are linked to hazards like hyperplasia, thrombosis, and infection [336]. Finally, in the case of patients suffering from CLI, it is of utmost importance to obtain rapid and short-term effects to prevent the loss of limbs, but the treatment of macrovascular disease may not bring about instant advantages.

Using cell therapy (for example, angiogenic stem cells or endothelial progenitor cells) [99] and administration of pro-angiogenic growth factors (delivered either as proteins or as protein-encoding genes) [72, 247] to induce and promote angiogenesis in ischemic tissues, considerable advancement has been made to prevent these surgical interventions. For example, clinical investigations indicated that intramuscular injection of hepatocyte growth factor (HGF) plasmids reduced ischemic ulcer area by more than 25 percent and increased blood perfusion in CLI patients (evaluated by an increment in the ankle–brachial index (ABI) from 0.46 to 0.59) [179]. Clinical advancements in CLI patient symptoms were also observed following intramuscular injection of VEGF plasmids and autologous bone marrow mononuclear cells (BM-MNCs). These injections led to enhanced perfusion (an enhancement of the ABI from 0.26 to 0.49) and reduced rest pain [251].

Despite these encouraging preliminary findings, more subsequent phase II and phase III clinical studies using angiogenic gene therapy did not show consistent effects in an anticipated way. Because of inefficient cellular transfection, directly injecting naked plasmids containing angiogenic genes is useless. It is possible to circumvent the inadequate transfection effectiveness using viral vectors to transfer the genes; however, this method raises safety issues due to the uncontrolled insertion into the host cell's genome. In addition, the incorporation of genes that result in the continuous production of angiogenic factors heightens the risk of developing cancer or pathological angiogenesis [85]. On the other hand, utilizing recombinant proteins is not as likely to result in such severe long-term safety concerns.

Nevertheless, growth factors typically have very limited circulation half-lives; hence, several injections are required to produce adequate and sustained growth factor concentrations at the ischemia location. Vascular leakage [264], tissue edema [221], and hypotension [52] are all potential undesirable consequences that might be brought on by receiving many injections of angiogenic growth factors. Currently used cell treatment procedures are plagued by several potential drawbacks, including poor post-transplantation vitality, poor cell retention, and restricted incorporation into host tissue [213].

### NPs for PAD detection

Magnetic resonance imaging (MRI) is a technology that does not involve any invasive procedures and has excellent soft tissue contrast represented by signal loss. It also has an unprecedented spatial resolution. Contrast agents form this signal following their buildup in the vasculature, and it is recognized by the MRI modalities, which provide information on functional characteristics, phenotypical characteristics, as well as molecular characteristics. Even though MRI is a widespread imaging method, studies have shown that it can provide pictures that are unreliable and inaccurate due to the presence of endogenous contrast agents. Exogenous contrast chemicals have been investigated to produce better pictures that can identify the localization and the characterization of atherosclerotic plaque [318]. For example, the expression of αvβ3 integrin is deficient in normal arteries, but it is extreme in atherosclerotic vessels. Since αvβ3 integrin is abundantly expressed in atherosclerotic vessels, it may be used as a biomarker to determine whether the vasculature has been damaged [97]. Using MRI, the visibility of damaged blood arteries in rabbits was improved by Gd-loaded liposome nanomaterials labeled with αvβ3 antibodies [250]. In addition, the expression of v3 vessels has been investigated using paramagnetic perfluorocarbon nanomaterials created explicitly to detect atherosclerosis in its earliest stages. MRI imaging showed that administering fumagillin-loaded paramagnetic perfluorocarbon NPs (175–200 nm) to rabbits resulted in reduced signals (about 3 percent), in comparison to the signals produced by untreated animals (about 18 percent) [293].

Researchers and physicians now have the opportunity to properly track the progression of PAD thanks to the deployment of MRI technology that uses nanostructures. This is made possible by including imaging agents, which are categorized as T1 and T2 shortening agents. The first method emphasizes the utilization of gadolinium (Gd^3+^), a contrast agent that can enhance the visibility of MRI images, whereas the second method emphasizes iron oxide NPs. T2 shortening agents such as magnetic iron oxide (Fe_3_O_4_, Fe_2_O_3_) contribute to this imaging technique by encouraging extravasation inside the vascular tissues, where they are then absorbed by macrophagic cells. This allows the T2 shortening agents to play a role in the imaging process [163]. Gd^3+^ is a positive contrast agent with seven electrons that are not paired. When it is present in water settings, it generates powerful magnetic fields that affect the pace at which water protons relax, which in turn results in a prolonged relaxation period (hence longer signals) [75, 131]. When linked to the vasculature of a disease, Gd^3+^ produces pictures that are more vivid when MRI detects the signal. This heavy metal molecule can act as a chelate, leading to significant problems with systemic poisoning. This issue is resolved by the synthesis of nanoprobes such as micelles, liposomes, and polymeric particles integrated into Mn2 + or Gd3 + to produce nanostructures with characteristics that offer brighter and more accurate T1-weighted MRI images [265]. When applied to damaged vasculature, including Gd3 + into nanomaterials will reduce the likelihood of the particles causing toxicity. In addition, NPs are praised for their simple surface modifications and their superior ability to target specific areas.

For instance, to specifically target human macrophages, gadolinium Gd^3+^-lipid nanomaterials were tagged with CD36 antibodies and employed in the experiment [153]. The MRI scans from this investigation demonstrated that the CD36-labeled lipid nanomaterials could detect and analyze atherosclerotic plaque. This information might be utilized to develop therapy choices better to avoid atherothrombotic occurrences. It has also been reported that the surface of micelles laden with Gd^3+^ can be easily customized to target the macrophage scavenger receptor (MSR) in animal models of atherosclerosis [8]. Because NPs can transport a greater quantity of contrast chemicals than a single tiny molecule while simultaneously decreasing their systemic toxicity, they are highly adaptable to in vivo imaging. T2 contrast agents are known as "negative contrast agents", because they generate dark pictures upon buildup at a disease site [278]. Due to the enhanced magnetic characteristics, iron oxide NPs are frequently employed as T2 agents [51]. It is essential to personalize these nanoprobes as much as possible, since the MRI signal can be affected by the surface charge, size, and shape. For example, the better tissue distinguishing capabilities of these iron oxides NPs are frequently related to their surfaces, typically covered by dextran or siloxane. This coating is located on the surface of the magnetite iron oxide core [149]. The nanomaterials' diameter is crucial, since the magnetic concentration rises as the NPs get massive when it comes to MRI applications. The detection of macrophages by the use of superparamagnetic iron oxide (USPIO) NPs (with a 5 nm in diameter core and a coated dextran of 10 nm thickness), monocrystalline iron oxide NPs (MION-47), and 3-T MRI has been utilized to quantify accumulated foam cells and to evaluate the progression of atherosclerosis [178]. In comparing the macrophage content of rabbit arteries that received rosuvastatin and those that did not get rosuvastatin, qualitative and informative photos of rosuvastatin-treated arteries showed a lower level of macrophage content after administration of MION-47 (controls). Amirbekian et al. used macrophytic foam cells to represent atherosclerosis in mice [8] and investigated these cells' absorption of MSR-conjugated USPIO NPs. After 24 h of delivery, an upregulation of signal intensity was approximately 79% higher than that of the controls (which were nontargeted micelles).

Investigation into the potential use of proangiogenic and proinflammatory oxidation-specific epitopes for PAD diagnosis has also occurred [37]. Compared to much more significant structures, NPs have an edge when it comes to in vivo detection, because they can correctly load massive levels of contrast while maintaining their capacity to traverse systemically inside the body. In work, designed lipid ultrasmall iron oxide particulates (LUSPIO NPs) that were coupled with single-chain (IK17) Fv antibodies and malondialdehyde [MDA]2 E06 were selective for the detection of PAD as well as atherosclerotic plaque that was at high risk for rupture. In addition, the MRI scans demonstrated a distinct nanoparticle uptake localization in the wounded sites compared to those of untargeted LUSPIOs (controls) [36]. A recent study used iron oxide nanomaterials coated with aminopropyltrimethoxysilane (APTS) to track cells implanted into the ischemic hindlimb of mice [333]. The localization and migration of the transplanted cells could be seen up to 4 weeks after the procedure using decreased signals from 3T MRI images. When employing SPIOs in conjunction with MRI, one of the drawbacks is that the local aggregation of NPs results in low-contrast pictures, which makes quantitative analysis more difficult.

### Novel NPs for peripheral artery disease (PAD) and PVD treatment

Due to insufficient blood flow to the limb extremities, CLI is an extreme condition of peripheral arterial disease (PAD). Patients diagnosed with CLI frequently experience excruciating immobility, delayed wound healing, cramping pain, amputation of the afflicted limb, cardiovascular issues, and, in severe cases, even death [268]. The standard treatment for CLI consists of surgical revascularization in addition to angiogenesis restoration with the use of growth factor therapeutic interventions [236]. Nevertheless, surgical revascularization is only appropriate for a small proportion of CLI patients and is linked to a significant perioperative death rate. The use of growth factors is also restricted due to the fact that they have low therapeutic angiogenic potential [197]. Growth factors have low bio-availability and they have problems with non-specificity. As a result, there is an immediate demand for the development of novel alternative novel therapeutic nanobiomaterials to treat CLI to circumvent the drawbacks that have been outlined before associated with the use of current techniques. During the course of the last few decades, a wide variety of research organizations have been engaged in the process of producing diverse pro-angiogenic nanostructures [205, 267, 290]. Zinc oxide nanoflowers (ZONFs) are one of the NPs that are believed to be among the most effective in terms of their ability to induce therapeutic angiogenesis. In previous research, researchers demonstrated that ZONFs stimulate angiogenesis of endothelial cells through activating Akt/MAPK/eNOS cell signaling pathways as well as by promoting the creation of ROS and nitric oxide [24]. Furthermore, they have also documented the medicinal efficacy of ZONFs to cure cerebral ischemia via their neuritogenic as well as neuroprotective capabilities, utilizing angio-neural cross-talk. In current research, designers investigate in depth the clinical efficacy of ZONFs in a rat hind limb ischemia model, an animal prototype that is analogous to CLI in humans [25]. This model was created by cutting off blood flow to the hind limb through the ligation of the femoral artery in the hind limb. The results demonstrated that the ZONFs administration could ameliorate rats' ischemia at an accelerated rate through helping to promote therapeutic angiogenesis to the ischemic locations. This was demonstrated by the fact that ZONFs were capable of being detected in the rats' plasma. In conclusion, the current research presents a novel nanomedicine method utilizing ZONFs for PAD treatment. Recent research has demonstrated that nanorods made of europium hydroxide can promote therapeutic angiogenesis, which in turn helps reduce the vascular toxicity caused by cadmium exposure [188]. This effect is seen in Wistar rats who have had myocardial ischemia. It is postulated that the proangiogenic europium hydroxide nanorods could be advantageous for the successful treatment of CLI, taking into consideration the significance of revascularization in the management of the condition. Therefore, the purpose of this work is to conduct an in-depth analysis of the therapeutic effectiveness of europium hydroxide nanorods by testing them out on a model of hind limb ischemia using Wistar rats. Ischemic rats that were given europium hydroxide nanorods exhibited better motility, greater blood supply to the ischemic limb, as well as higher expression of angiogenic factors. In addition, toxicology and pharmacokinetic investigations of europium hydroxide nanorods in mice reveal that they are harmless, suggesting that it may be possible to use these nanorods in a realistic clinical setting to treat CLI.

The initial step in treating PAD is to employ balloon angioplasty to mechanically remove any blockages throughout the blood vessel [266]. The vascular re-narrowing or restenosis that results after surgical intervention can be prevented by coating the balloons with antiproliferative agents, although existing drug-coated balloons that release chemotherapeutic drugs like paclitaxel have in certain cases demonstrated higher mortality over the long term [152]. Ioana Craciun et al. developed a novel drug-coated balloon by employing a polymeric nanodelivery method [50]. This would allow for the prolonged release of polyphenols, which not only prevent restenosis but also have a lower level of toxicity in comparison to chemotherapeutic medicines. The development of PLGA NPs containing entrapped quercetin, a dimethoxy quercetin (rhamnazin), and quercetin covalently bonded to PLGA, in addition to quercetin, led to the discovery of these compounds. Using an ultrasonic technique, balloon catheters were covered with polymeric NPs. Across all of the NPs delivery methods that were put to the test, the ones that had quercetin connected to them via a covalent bond delivered the longest lasting release over the course of a week. In comparison to previous nanoformulations, the degree to which these particles attached to cells was much lower, yet their adhesion was resistant to being removed by washing. In addition, these NPs had the strongest capacity to inhibit cell proliferation. In addition, their attachment was not affected when the cells were cultured in calcifying circumstances, despite the fact that calcification is normally a state that occurs in PAD tissue and represents a condition that hinders medication delivery. In addition, the ultrasonic coating process produced a covering that was consistent all across the balloon. As a result, the polymeric NPs with covalently bonded quercetin that were created in this study have been offered as a potentially useful platform for reducing the risk of restenosis following angioplasty.

A naturally occurring phenolic substance with antioxidant effects is salvianolic acid B (SAB). On the other hand, the limited applicability of hydrophilic SAB may be due to its low bioavailability [244]. Boronic ester-based NPs can act as ROS-responsive vehicles that can quickly and selectively release drugs at the site of injured tissue [18]. Patients diagnosed with PAD have been treated with the polysaccharide dextran in an effort to reduce platelet aggregation [291]. Laponite is a kind of nanoclay that is produced in a lab and has found widespread use in the medical field [73]. As a carrier for SAB distribution, laponite hydrogels incorporating ROS-responsive dextran-based NPs were produced for the study and used as a part of the research [45]. Under conditions of oxidative stress, the boronic ester modified dextran NP had a spherical shape and had a diameter of 195.3 nm. It could be hydrolyzed fully in under 9 min. The treatment of HUVECs with SAB at a concentration of about 100 μM was most effective. The findings showed that SAB-loaded NPs were uniformly distributed throughout the lattice of laponite hydrogels. When the shear load was removed, the rheological behaviors of laponite containing SAB-NPs displayed shear-thinning qualities and an enhancement in fluidity. Laponite-containing SAB-NPs with sustained drug release capabilities were capable of scavenging ROS as well as restoring HUVECs from oxidative damage through a reduction in the inflammatory level (IL-6, IL-1β, I IL-1α, MMP-9, and TNF). In-vivo testing has shown that the hydrogels that have been produced are compatible with living organisms. The findings indicate that the newly produced laponite containing SAB-NPs can have therapeutic applications for PAD treatment.

## Angiogenic therapy based on protein delivery using NPs

In the research and clinical investigations conducted over the past decade, the central emphasis has been on utilizing pro-angiogenic genes or growth factors to stimulate angiogenesis in ischemic tissues [243, 317]. Because they have such a short half-life throughout the bloodstream, bolus injections of growth factors rarely produce satisfactory clinical results (in several minutes) [139]. However, the formation of steady neovascularization requires significant growth factor doses to be delivered either intramuscularly or intraarterially [47]. This is because elevated sustained rates of angiogenic signaling are needed for the process. Utilizing nanosized structures is one way to overcome this shortcoming of protein-based treatments for medical conditions [140]. The encapsulation of growth factors within nanocarriers facilitates targeted delivery towards the ischemic tissue, safeguards them from unexpected degradation, and facilitates their release in a controlled manner. As a result, the therapeutic effects of the growth factors are increased, and the patient may require lower doses of drugs.

Many different nanostructures have been utilized to transport growth factors into ischemic tissues. These NPs include poly(lactic-co-glycolic acid) [80], gold NPs [125]; R [218], graphene oxide [334], and PLGA: poloxamer hybrid nanostructures [53]. It has been demonstrated that when intravenous injections of NPs (less than 200 nm in size) are given to mouse models, the nanostructures preferentially aggregate in ischemic limbs instead of healthy limbs [200, 257]. Even though no targeting molecules or particular antibodies were included in the nanostructures, this was the case. Ischemic tissues, such as cancerous tissue, tend to generate angiogenic factors, which enhance the permeability of blood vessels and lead to preferential nanoparticle accumulation. The term "enhanced permeability and retention" (EPR) describes this well-known phenomenon that may be found in many environments [104]. More interestingly, graphene oxide NPs coated with VEGF exhibited a remarkable increase in targeting effectiveness compared to empty nanostructured materials [98, 121]. Hence, it is suggested that VEGF coated on the particle surface would behave as a therapeutic reagent and be employed as a targeting moiety. ThiIt must be considered that the overexpression of VEGF receptors on the cell surface found in ischemic areas. Because of their inherent targeting potential, NPs that contain growth factors can be delivered intravenously, which is a method that is both less intrusive and more convenient than injecting them directly into the muscle.

Due to the adaptability of NPs, it is possible to modify both their physicochemical properties to construct a release kinetic profile that is unique to them. To obtain a continuous growth factor concentration at the ischemia region without the demand for numerous injections as well as to allow for the stability of newly created capillaries, the zero-order release kinetics profiles are recommended. This allows the most significant amount of time for the growth factor to enter the bloodstream. It is possible for Dextran-co-gelatin nanomaterials to reach near zero-order release, with 69 percent of VEGF being released over 10 days in vitro [303, 304]. In vitro testing showed that mesoporous silica NPs constantly produced bFGF for over 20 days, with half of the total released in the first 8 days [326]. In addition, PLGA nanomaterials demonstrated a burst release pattern in vitro, with 70 percent of the encapsulated VEGF being unleashed within two days[79]. Despite this, it is possible that the in vitro release profiles may not accurately reflect the in vivo release kinetics because of the biochemical complexities of the in vivo conditions. Tracking the levels of growth factors in blood samples collected from the ischemic region is expected to offer further insights into pharmacokinetics. Furthermore, the functionality of growth factors cannot depend on the NPs being encapsulated; otherwise, the growth factors can be coupled on the surface of the nanomaterials. It has been established that maintaining the bioactivity of VEGFs and stimulating the development of endothelial cells to generate new blood vessels can be achieved by combining VEGFs with gold nanomaterials covalently using gold–thiol interactions [125, 130]. In this manner, the undesirable disintegration of growth factors can be slowed down, which will increase the levels of active growth factors in the ischemic region.

In animal models, the administration of growth factors through NPs has produced promising outcomes. In a rabbit ischemic model, the restoration of blood perfusion in ischemic tissue to 85 percent of healthy tissue was achieved with an intramuscular injection of dextran-co-gelatin nanomaterials encapsulating 1 mg of VEGF in total [303]. While a bolus injection of the relatively similar level of free VEGF could not lead to any considerable enhancements, intravenous injection of only 3 µg of VEGF conjugated on gold nanomaterials improved ischemic limb blood perfusion by 1.7-fold, achieving over 90 percent blood perfusion of healthy tissues [125]. Doppler scanning revealed that the administration of 3 µg of VEGF via graphene oxide nanostructures led to an increase in blood perfusion that was 1.5 times greater than before. Liposomal co-delivery of FGF-2 with syndecan-4, an essential regulator of FGF-2 signaling, improved the cellular signaling responses to FGF-2, which led to higher FGF-2 uptake and an enhancement of 80 percent in blood flow in comparison to delivery of FGF-2 alone [105]. This method improved the density of tiny arteries, but it also greatly raised the number of large vessels in ischemic muscle. In particular, growth factor therapy's efficacy can be reduced if the patient also suffers from co-morbidities, such as diabetes and hyperlipidemia. In addition, administering syndecan-4 proteoliposomes combined with FGF-2 was shown to enhance the efficiency of FGF-2 in diabetic mice, therefore, partially reversing the growth factor resistance that was present [55].

Even though the findings of the preclinical experiments have been encouraging, more in-depth research is still necessary to completely grasp the benefits and drawbacks of nanosized protein delivery. The vast majority of research provided evidence that the treatment was effective in neovascularization and acute ischemia models. In addition, there is no doubt that fast angiogenesis is crucial for CLI patients. In contrast, investigators infrequently have, if ever, assessed the long-term potential of the newly regenerated vascular system. In particular, it is feasible that the neovasculature might effectively regress over time if the availability of exogenous growth factors is depleted [19]. In addition, while angiogenesis is a complicated physiological process requiring the coordination of multiple different types of cells, the approaches now being used are relatively straightforward and supply no more than one or two components at most. To further improve the angiogenic benefits, multiplexed nanomaterials that contain numerous enzymes and growth factors with sequential release and optimal stoichiometry can be produced [48].

## Angiogenic therapy based on Gene delivery using NPs

Proteins are rapidly biologically active and, therefore, can effectively act on membrane receptors of their targeted cells, which is one benefit of protein delivery over gene delivery. On the other hand, Genes require a complicated delivery system within the cells and translocation into the nucleus to be transcribed and translated before they can be expressed. Meanwhile, the supplied proteins will be progressively consumed, but genes may make vast amounts of proteins on their own, making it possible to achieve sustained concentrations of growth factors without frequent injections. Therefore, using an appropriate vector that can transport genes into cells is essential to accomplishing any gene therapy. The CRISPER/Cas9 system has recently brought about a revolution in gene editing and gene delivery. This system can either inhibit or activate specific genes. However, for this system to work, an adenovirus must be introduced into the cell to transport the Cas9 protein and any related synthetic transcription factors or RNA [164]. It is standard practice to utilize adenoviral or viral vectors in investigations due to the excellent transfection effectiveness they offer. However, these vectors are also connected with several potential safety risks, including the insertional mutagenesis of host cells or the induction of immunological responses. These risks have been the primary barrier to successfully translating this innovation into clinical settings [281, 282, 284].

On the other hand, nonviral vectors, including polymeric materials or lipids, are appealing, because they are less hazardous than viral vectors and may be designed in a wider variety of ways [337]. However, in comparison to viral vectors, they are often less efficient. To accomplish efficaciously as well as successful gene delivery, it is necessary to bypass a large number of barriers, both those found inside cells and those found outside cells [232]. The nanocarrier needs to prevent the DNA from being degraded by serum DNases, can travel through the bloodstream, target the specific cell type at the targeted location, enter the targeted cell through internalization, and avoid being taken up by the reticuloendothelial system (RES) or the phagocytic cells, escape from the endosome into the cytoplasm, and translocate into the nucleus and eventually release the payload. In addition, other design goals, such as simple production, minimal toxicity, and a cheap cost of synthesis, need to be accomplished [199].

The preclinical assessment of nanoparticle-based gene delivery techniques to treat peripheral ischemia has produced positive results. In a rabbit hindlimb ischemia model, magnetic gelatin nanospheres complexed by VEGF plasmid (5–20 nm) were intra-arterially administered and then magnetically directed towards the ischemic region. This resulted in a fifty percent improvement in the density of blood vessels as compared to nanospheres that were not complexed with plasmid [107]. In a mouse model of hind limb ischemia, intramuscular injection of PLGA nanomaterials containing VEGF plasmid led to a 2.6-fold improvement in capillary density compared to the untreated group. Meanwhile, injection of polyethyleneimine–DNA NPs only resulted in a 1.4-fold improvement in capillary density [117]. This disparity could be partially explained by the fact that PLGA NPs have greater transfection effectiveness than polyethyleneimine, as demonstrated by the fact that VEGF expression is higher in mouse limbs. Furthermore, polyethyleneimine was cytotoxic and caused a cell apoptosis rate four times higher than that caused by PLGA NPs. The use of ultrasound in conjunction with nanocarriers is yet another potentially fruitful approach [186]. Ultrasound can be used for image processing and monitoring nanostructures by utilizing NPs designed to devour gas bubbles. Ultrasound can also be used to enhance the transportation of molecules inside cells by acting as an intracellular transporter. As an echo-contrast, perfluoropropane gas is encapsulated in 200 nm PEG-liposomes, transporting the bFGF plasmid more effectively than the naked plasmid (less than 40%) [187]. Similarly, the intravenous injection of PEGylated-perfluoropropane gas liposomes packed with a negative regulator of VEGF inhibitors, miRNA-126, improves blood circulation inside the ischemic limb in a mouse by thirty percent [67].

## Stem cell angiogenic therapy using NPs in cardiovascular disease

Previous research has demonstrated that genetic engineering can help promote stem cell therapy for heart disease [93, 170]. This achievement is accomplished by introducing therapeutic genes (such as antiapoptotic and proangiogenic genes) into engineered stem cells through gene vectors to enhance their paracrine secretion and prolong their survival [58, 261]. Traditional vectors have restricted use because of the immunological reactions they elicit and the minimal gene volume they carry. When transmitted to stem cells, nanoparticle-based genes with specific biocompatibility can have a greater gene delivery effectiveness, leading to increased differentiation and cell survival in ischemic myocardium. Inorganic nanostructures have simple fabrication specifications and induce minimal cytotoxicity, which can be used with blended vectors. Hence, the liposome can protect genes from being deteriorated or from attaching non-specifically [63]; polymeric materials can enhance effectiveness [63], help decreases cytotoxic effects, as well as strengthen targeting specificity; and inorganic nanomaterials can enhance targeting specificity [287]. Endocytic pathways involve the processes by which their contents are taken within the cell. These routes include micropinocytosis, caveolae-mediated endocytosis, clathrin-mediated endocytosis, and phagocytosis [39]. In their research, placental growth factors were delivered in a targeted way using chitosan–alginate NPs. This method has the potential to meet the objective of constantly releasing placental growth factors and enhancing heart function at the site of acute myocardial infarction [31]. A further investigation transfected mesenchymal stem cells (MSCs) with molecularly organic–inorganic blended hollow mesoporous organosilica NPs that had a surface conjugated with polyethyleneimine and packed with the hepatocyte growth factor gene. The researchers discovered that the paracrine activity of the hepatocyte MSCs transfected growth factor was improved, which limited the myocardial cell apoptosis as well as enhanced angiogenesis in the rat model [335].

## Age-related macular degeneration and diabetic retinopathy

As one of the most common causes of blindness, diabetic retinopathy is a secondary consequence of Type 2 Diabetes or Diabetic Mellitus. Diabetes affects the whole neurovascular areas of the retina, causing gliosis, angiogenesis, neurodegeneration, fibrosis, neuroinflammation, and edema to occur continuously [1]. When the vasculature alters, there is a detectable alteration in visual perception, which eventually results in blindness. When exposed to hypoxic conditions, the antioxidant VEGFA is elevated, and this upregulation performs a critical function in developing diabetic retinopathy. In addition, the enzyme matrix metalloproteinase-9 (MMP-9) has been linked to the initiation and progression of diabetic retinopathy [208].

Another consequence of pathological angiogenesis is age-related macular degeneration (AMD), which affects the eye's retina. AMD can be divided into two categories. Those who have dry AMD (marked by yellowish deposits in the macula) are known as dry type AMD; those who have wet AMD (defined by distinctive choroidal neovascularization) are known as neovascular or wet type AMD [315].

The therapeutic options used for disorders that damage the vascular system in the posterior eye include laser photocoagulation and repeated intraocular injections. In addition, it causes side effects, such as the deterioration of healthy tissue. It was suggested that one of the therapeutic options be "introducing protein medicines," but it had specific problems, such as pharmaceutical instability caused by protease activity following administration of medicines. As a result, innovative therapeutic procedures were required to overcome these disadvantages. Accordingly, in an attempt to generate additional therapeutic techniques for ocular illnesses, the effectiveness of various candidate nanomaterials, including those with inherent antiangiogenic properties or those with the potential to transport drugs, growth factors, as well as other therapeutic agents to particular tissue areas, has been evaluated by several groups [7, 32]. Gold nanostructures have anti-angiogenic effects in conjunction with their superior biodegradable, electrical, and molecular-recognition features [311]. VEGFR2 in HUVECs has been shown to undergo nano-structured remodeling, which in turn suppresses angiogenesis [202]. Gold NPs have also been shown to inhibit cell migration induced by VEGF in retinal endothelial cells by adversely influencing the phosphorylation of eNOS and Akt in these cells. VEGF treatment of retinal endothelial cells has also been shown to inhibit their growth by decreasing Src signaling pathways [125, 126].

A proteolytic portion of plasminogen with 80 amino acids known as Kringle 5 (K5) has been proven to be particularly efficient in inhibiting endothelial cell proliferation [41]. In the oxygen-stimulated retinopathy model, K5 can also slow the progression of retinal neovascularization induced by ischemia. However, it had the disadvantage of having a short lifetime [324]. K5-containing NPs were created by encapsulating an expression plasmid of K5 using PLGA polymer to make nanocarriers, which were efficient in inhibiting VEGF expression and attenuating retinal neovascularization ischemia-stimulated retinal vascular leakage in an oxygen-induced retinopathy rat model [206]. Biodegradable nanostructures packed with fenofibrate are especially effective in the targeted treatment of neovascular AMD and diabetic retinopathy. In addition to being a PPAR-α (peroxisome proliferator-activated receptor α) agonist, fenofibrate is also helpful in the treatment of diabetes. The vasculature permeability in the retina of diabetic rat models was observed to decrease 8 weeks following the administration of fenofibrate-loaded nanostructures by a single intravitreal injection. As an additional benefit, the development of retinal leukostasis was prevented, as well as the expression of ICAM-1 (intercellular adhesion molecule 1) and VEGF was reduced [216]. Octreotide, a somatostatin analog, is a well-established anti-angiogenic and neuroprotective medication that works by inhibiting VEGF production. It has been proposed that the intra-ocular administration of octreotide in combination with magnetic NPs will increase the bioavailability and the half-life of octreotide if accomplished in a controlled environment. Nanomaterials were targeted to capillary endothelial cells in the retina by Polliner et al. who indicated that quantum dots modified by Cyclo (RGDfC) particularly attach to the α_v_β_3_ integrin receptors on the endothelial cells and also that the cellular absorption caused by receptor affinity results in the accumulation of the designed nanostructures within both the intraretinal capillaries and choriocapillaris [212]. PLGA nanomaterials coupled with a linear RGD peptide having the ability to bind integrin were employed by Luo et al. as a nanovehicle for the transportation of recombinant Flt23k interceptor plasmid, including binding domains to VEGF. After intravenous injection, the nontoxic RGD-functionalized nanoparticle delivering platform was explicitly targeted to the choroidal neovascularization areas, demonstrating good vision restoration in mouse AMD and primate models [160]. Celecoxib is an inhibitor of cyclooxygenase-2 with anti-angiogenic and anti-inflammatory effects. In animal studies, poly (ortho ester) NPs loaded with celecoxib were proven to be highly efficient against diabetic retinopathy and AMD [201]. It has been shown that interleukin-12 has anti-angiogenic properties by lowering the concentrations of MMP9 and VEGFA throughout the bloodstream [228]. Zheng and colleagues mixed IL-12 with PLGA nanomaterials and showed that this combination was more effective in inhibiting the expression of MMP9 and VEGFA in the diabetic retinopathy mouse model and endothelial cells of rats. Furthermore, the intraocular delivery of these nanoparticles in a diabetic retinopathy mouse model demonstrated decreased retinal injury [321].

## Diabetic wounds

Some variables contribute to diabetic wounds taking longer to heal. First, an elevated level of blood sugar causes the arteries to become stiff and the blood vessels to narrow, which inhibits the flow of oxygen and nutrients necessary for the body's natural healing process [305]. Second, diabetic patients have a higher chance of developing PAD, a condition that reduces the amount of blood that can flow to the lower extremities. Consequently, they often suffer from poor or reduced blood circulation. Most commonly, PAD is a condition that most commonly affects those who have chronic wounds, most notably diabetic foot ulcers. Third, an inadequate blood flow throughout the body is the root cause of peripheral neuropathy, often known as nerve injury. This disorder causes a decrease in the flow of oxygen and nutrients to the tissues and nerves in the extremities, which in turn causes the nerves in the surrounding region to be damaged and a reduction in insensitivity to pain, temperature, and touch. Finally, diabetes makes the immune system less effective by reducing the body's capacity to send white blood cells to combat germs. As a result, wounds that have been diabetic-affected are more likely to get infected. Consequently, diabetic wounds frequently progress into infections, such as diabetic foot ulcers that do not heal. Regular checks of the feet, protection against infection, and maintaining sound management of diabetes by tracking cholesterol levels, glucose levels, and blood pressure can all help avoid diabetic wounds [138].

It was reported that after applying Graftskin, a high rate of wound healing occurred for a maximum length of 4 weeks without any significant adverse effects[277]. There is a lack of angiogenesis and vascularization in diabetic individuals, which are biological elements that contribute to delayed wound healing. It is possible to lower the amputation rate by correcting these defects; hence, studies have sought to accomplish this objective by focusing on several pathophysiological pathways. New treatments for diabetic wounds, such as skin substitutes, negative pressure wound therapy, hyperbaric oxygen, wound dressings with the inclusion of growth factors, and bioengineered tissues, have been developed thanks to advances in technology [210]. These treatments include skin substitutes, negative pressure wound therapy, and hyperbaric oxygen. For instance, topical VEGF was used to treat diabetic wounds because of its potential to maximize epithelization, matrix deposition, proliferation, and release of platelet-derived growth factor (PDGF)-B and fibroblast growth factor-2. This treatment was successful because of VEGF's unique properties [74].

According to the reports, the NPs stimulated angiogenesis and prevented bacterial infection in diabetic wounds. This resulted in rapid wound healing rates and re-epithelization, exhibiting enhanced granular tissue formation and increased VEGF expression [286, 287]. It has been demonstrated beyond a reasonable doubt that gold NPs have anti-inflammatory properties, and when combined with antioxidants, these NPs can hasten the healing of cutaneous wounds [211]. An injectable adhesive thermosensitive multipurpose polysaccharide-based dressing (FEP) with prolonged pH-responsive exosome release was manufactured by Min Wang et al. to stimulate angiogenesis and diabetic wound healing [281, 282, 284]. The FEP dressing possessed multifunctional characteristics, such as self-healing behavior, fast hemostatic ability, effective antibacterial activity/multidrug-resistant bacteria, good UV-shielding performance, and suitable tissue adhesive. These properties allowed the dressing to serve a variety of purposes. In vitro studies have shown that adding FEP@exosomes (FEP@exo) may significantly improve endothelial cell migration, proliferation, and tube formation. The in vivo results of a diabetic full-thickness cutaneous wound model showed that using a FEP@exo dressing sped up the wound's healing process by boosting the wound's angiogenesis process.

Likely, the deposition of collagen, the creation of granulation tissue, remodeling, increased cell proliferation, and re-epithelialization are the factors that contribute to the rapid healing with less formation of scar tissue and skin appendage regeneration. According to the findings of their study, including bioactive compounds into dressings with several functions should be highly able to promote adequate wound healing in patients with diabetes and other conditions linked to vascular impairment. Microvascular responses to AZD8601, a modified mRNA expressing VEGF-A, were investigated in vivo by Naidi Sun et al. longitudinally and thoroughly. Using multiparametric photoacoustic microscopy, they demonstrate that intradermal injection of AZD8601 formulated in a biocompatible vehicle leads to intense, prolonged, and dose-dependent vasodilation neovessel formation and blood flow upregulation. These effects are in striking contrast to those caused by recombinant human VEGF-A protein, a non-translatable version of AZD8601, and citrate/saline vehicle. In addition, they tested the bioactivity of AZD8601 using a mouse in an in vivo model of diabetic wound healing. They demonstrate that sequential dosing of AZD8601 increases vascularization and tissue oxygenation of the wound bed, resulting in rapid re-epithelialization during the early phase of diabetic wound healing. This was accomplished using a tissue oxygen sensor based on boron NPs [256].

## Pro-angiogenic factors and anti-angiogenic therapy for eradication of tumors

The term "angiogenic" refers to the process of new blood vessels forming from the vascular system that already exists. Wound healing, ovulation, and embryonic development, are just a few of the numerous processes in which it plays an important part. In addition, it is a crucial factor in the pathogenesis of a great number of disorders (including cancer metastasis and arthritis) [230, 231, 241]. Because nutrients and an appropriate oxygen supply are necessary for the survival of metastatic cells and the development of tumors, metastatic cells need to remain in close proximity to blood capillaries to maintain direct contact with the circulatory network [157]. During the progression of cancer, tumor cells and also the stromal cells that are related to tumors trigger an angiogenic switch through the continuous secretion of pro-angiogenic factors, which then encourage the proliferation and migration of endothelial cells. This results in the formation of new blood vessels during cancer development. This process also causes the development of disordered and immature vasculature with damaged endothelial cell junctions, which is related to tumor interstitial fluid pressure, neo-vessel permeability, and fragility [155]. When there is an imbalance between proangiogenic factors, such as bFGF, VEGF, insulin-like growth factor 1 (IGF-1), angiopoietin-2, and hepatocyte growth factor (HGF); and antiangiogenic factors, such as angiopoietin-1 and thrombospondin-1, the development of blood vessels within tumors can occur [84]. Pro-angiogenic activity can be exhibited by fibroblast growth factors (FGFs) when they attach to surface receptors of endothelial cells, including heparan sulfate proteoglycans, tyrosine kinase receptors, and integrins. Because of the close relationship that exists between FGFs and angiogenesis, researchers believe that lowering FGF concentrations might be an effective strategy for preventing the growth of tumors [68, 214]. One of the better-known pro-angiogenic factors is FGFb [319]. In addition, the suppression of proangiogenic factors, such as VEGF, FGFs, and the abovementioned factors, might result in an antiangiogenic impact or behavior of NPs, especially once they are involved in neovascularization. This can bring about the antiangiogenesis capabilities of NPs [230, 231].

Judah Folkman made a ground-breaking discovery in 1971 about the importance of angiogenesis throughout cancer development[71]. This discovery ushered in a new age of cancer research focused on targeting proangiogenic factors towards cancer therapies. It has been demonstrated that tumors are unable to grow larger than 2 mm in diameter unless they get a continuous supply of nutrients and oxygen through angiogenesis [108], which has been demonstrated. As a result, it has been argued that one of the most critical approaches to cancer treatment is to avoid neovascularization as well as target proangiogenic factors. Tumor microenvironment angiogenesis differs from healthy angiogenesis, since it is distinguished by developing a premature, leaky vascular system and its blood vessels, which results in a constant inflammatory response [15]. This occurs primarily due to the enhanced production of pro-angiogenic agents like integrins, angiopoetin, VEGF, and other similar proteins, and these molecules are being targeted in anti-angiogenic treatment. Nevertheless, the release of additional pro-angiogenic factors outweighed the efficacy of anti-angiogenic drugs that target VEGF, like Bevacizumab, authorized by the Food and Drug Administration (FDA) [20, 88]. As a result, combining treatments using several anti-angiogenic substances was more effective in overcoming resistance to angiogenic monotherapies.

Using nanostructures as a medium to carry several medications targeting pathways and molecules linked with tumor angiogenesis can revolutionize cancer treatment [4, 275]. Biochemical encapsulation or conjugation of medicinal medicines onto NPs is the most common loading method [20]. The distribution of drugs by nanomaterials can occur in either an active or a passive manner. EPR is a phenomenon that occurs in the appearance of permeable blood vessels throughout the proximity of malignancies that aids the passive extravasation of NPs having sizes less than 200 nm into the malignant cells, with such nanostructures being removed by the liver subsequently [296]. Furthermore, the presence of inadequate lymph drainage aids in preserving nanostructures at the location of malignancies, which strengthens and encourages the long-term administration of drugs. Nanomaterials coupled with Doxorubicin [44] or smaller chemical blockers of angiogenesis [87] have been shown to concentrate inside the tumor microenvironment through the EPR mechanism, resulting in the stopping of tumor angiogenesis as well as the development of cancerous tumors in animal models. Furthermore, it has been observed that caplostatin (TNP-470), as an angiogenic blocker, accumulates preferentially inside the vascular systems related to tumors as a result of the EPR effect, hence inhibiting cancer-related vascular hyperpermeability. The active target of vasculature using nanomaterials is accomplished by the presence of ligands upon the exterior of the nanomaterials [239]. It is anticipated that the ligands will preferentially attach to receptors that are abundantly expressed on cancer cells and also on cancer-related endothelial cells, like α_v_β_3_ integrins, and VEGFRs [20, 302].

**Fig. 2:**
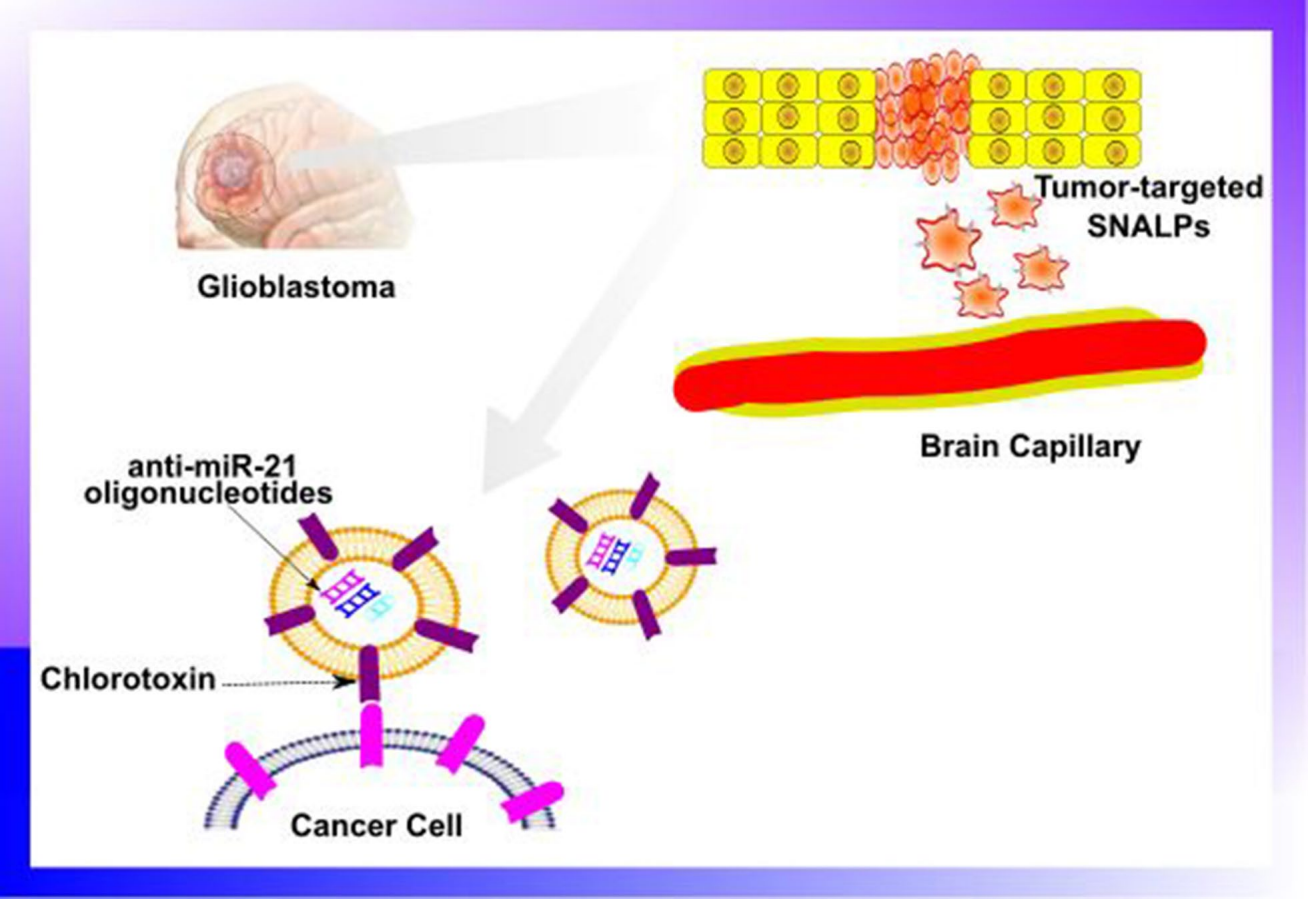
Intravenously administered anti-miR-21 oligonucleotides formulated with chlorotoxin (CTX)-coupled (targeted) stable nucleic acid–lipid particle (SNALP) innovation accumulates selectively inside of brain tumors as well as facilitate effective miR-21 silencing, resulting in enhanced mRNA and protein levels of its target RhoB, with no evidence of systemic immunogenicity

Nanostructures have targeted several distinct miRNAs, and their therapeutic effectiveness has also been investigated. The use of anti-miR-21 nanoplatforms in patient populations having glioblastoma, for example, has been shown to reduce the expression of the miR-21 targeting gene RhoB at both the protein and mRNA levels in individuals with this cancer type (Fig. 2). Furthermore, anti-miR-21 treatment by NPs has been shown to reduce tumor development, trigger apoptotic cell death, and increase the rate of survival in experimental models of cancer [49]. Exosomes are NPs based on lipids essential for the transportation of macromolecules, such as proteins and RNA among cells. MiR-23a-contained exosomes successfully induce angiogenesis in both the in ovo xenograft model and the CAM model by controlling the SIRT1 gene expression [255].

Several metallic NPs, such as silver and gold NPs, are beneficial in anti-angiogenic treatments. The ability of gold NPs to attach to the binding domains of heparin of several growth factors, including bFGF and VEGF165, has been demonstrated. This adherence results in morphological alterations of the growth factors that are linked with diminished functionality. The phosphorylation of VEGFR2 was negatively regulated by VEGF inhibition mediated by gold NPs, which was previously discovered. Moreover, it was shown that the dimension of gold NPs significantly impacted their suppressive impact on heparin-binding growth factors (HB-GFs). Gold NPs with a diameter of 20 nm exhibit the most excellent suppressive activity. In addition, it was discovered that gold NPs with an uncoated exterior were required for the inhibitory impact on HB-GFs to occur. Furthermore, gold NPs have been shown to suppress the MAPK pathways throughout cancerous cells, which results in the suppression of the transition from epithelial to mesenchymal and, as a result, the prevention of metastasis mechanisms from occurring [11, 12]. Migration, cell proliferation, and tube development induced by VEGF in bovine retinal endothelial cells (BRECs) have been inhibited by silver NPs. According to some reports, it can also prevent the growth of vessels in the matrigel plug assay technique. According to the findings, the anti-angiogenic impact of silver NPs involves the downregulation of the PI3K/Akt pathway. Another study found that silver NPs have an anti-angiogenic impact by suppressing the transcription factor HIF-1 dose-dependent [309].

## Rheumatoid arthritis

Effective abrogation of rheumatoid arthritis requires actively targeted nanostructures to surmount at least three obstacles to obtain excellent results. For example, when nanocarriers were administered intravenously, the reticuloendothelial system was the first obstacle they encountered. Opsonization reduced the effectiveness of nanomaterial transportation to inflamed areas by reducing the circulating durations throughout the bloodstream. The following major limitation was an irritated endothelium of the circulatory system. Endothelial cells' secretion of matrix-degrading enzymes in response to inflammatory stimulation allowed the basal membrane to be digested. The cells multiplied and migrated to create primitive nanotubes, which later progressed into angiogenesis[66]. Angiogenesis can be prevented by targeting circulating NPs to the aberrant vascular system; however, it can also be relieved by moving nanomaterials through endothelium to reach the joint cavity and reduce additional rheumatic complaints like this inflammatory response. The third barrier is that the ligands coupled with the nanostructures ought to be selective, which means that only the matching receptors expressed by the targeted cells on their surfaces should be used to minimize adverse reactions to the greatest extent possible.

When taken together, the following characteristics of an optimal active targeted nanoparticle for rheumatoid arthritis therapy must be met: sufficient diameter (varying from 10 to 100 nm), as well as surface charge (anionic or neutrality), are required, as is PEGylation to extend the circulation period, and appropriate ligands are required for modification. In particular, nanomaterials of sufficient dimension might evade both filtrations by the kidneys (if their size is greater than 10 nm) and liver trapping (if their size is less than 100 nm) [54]. Aside from that, enhanced angiogenesis causes endothelial cell layers to become disjointed and hyperpermeable, allowing nanostructures having a diameter of roughly 100 nm to, at least partially, infiltrate the inflamed endothelium via the passive route, allowing for the delivery of drugs.

To this day, nanostructures treated by poly(ethylene glycol), i.e., PEGylation, remain the most extensively employed approach for effectively preventing opsonization of NPs. To achieve the brush structure, Leaf Huang proved that an optimum PEGylated nanocarrier should be adjusted by greater than 8 mol percent of polyethylene glycol(PEG) to offer complete coverage of the nanocarriers' surfaces [146]. While it is possible to manufacture durable, PEGylated NPs with a brush shape in principle, it is challenging to do so while retaining the lipid membrane’s integrity. Furthermore, a significant concentration of PEG can interfere with the attachment or absorption of nanostructures in targeted cells, resulting in a counterproductive impact. Aside from the densities, various chain lengths or topologies of PEG, and diverse covering techniques, such as physical or covalent adsorption, might all impact the PEGylation properties of the material.

Researchers Kyung-Hwa Yoo and colleagues created PLGA nanomaterials loaded with Methotrexate (MTX), then deposited a 15 nm gold (Au) film onto the nanomaterial monolayer, resulting in the half-shell architecture formation [143]. As the active target, MTX–PLGA–gold was used first, followed by the conjugation of cyclic RGD peptide on the gold surface. Afterward, the same group created a comparable nanomaterial by substituting the gold film on the nanomaterial monolayer with an Au/Fe/Au complex film, then applied it to the nanomaterial monolayer [123, 124, 127]. Mice experiencing collagen-induced arthritis (CIA) were treated using nanomaterials, and the amount of gold-based nanofilms accumulated inside the inflamed joints was evaluated to determine the number of nanostructures. When comparing the RGD modified group to the non-RGD modified group, a greater quantity of gold-based nanofilms was discovered within the inflamed joints of the RGD modified group, which was primarily attributable to the attachment of the RGD peptide to the αvβ3 receptors.

Given that active macrophages overexpress the scavenging receptors and that dextran sulfate (DS) is an effective ligand for the class A scavenger receptor, self-assembled NPs based on polycaprolactone (PCL) as a hydrophobic block and DS as a hydrophilic block were designed [129]. As demonstrated in its in vivo biodistribution research, DS-b-PCL NPs were shown to aggregate predominantly in the inflamed synovium of CIA mice. Take notice that this study just supplied a transporter and that no medicinal medication was encapsulated. The Fc receptors were targeted in another study, which employed poly (lactic/co-glycolic acid) nanostructures coupled with anti-CD64 antibodies to co-capsulate the MTX and SPIONs (superparamagnetic iron oxide NPs) [180]. This multipurpose nanostructure includes SPIONs as contrast agents for MRI detection, MTX as a therapeutic medication, and an anti-CD64 antibody as the active target. Even though these NPs were well defined in vitro, the scientists could not give in vivo data. A further achievement was the formulation of albumin nanomaterials loaded with etoricoxib coupled with folic acid, which was effectively used to address active macrophages [30]. Because of their antiangiogenic and anti-inflammatory properties, nanomaterials have been used as a medicinal therapy for rheumatic disorders [96]. A compound of hyaluronate–gold NPs and tocilizumab featuring simultaneous targeting properties was developed partly due to this characteristic. As demonstrated by Lee et al. gold NPs can bind actively to VEGF, while tocilizumab, the initial therapeutic antibody against IL-6 signaling, possesses the potential to target the IL-6R[141]. Researchers utilized the CIA model mice to establish that this compound had a therapeutic impact.

## Factors affecting the behavior of nanomaterials on vascularization and angiogenesis

### Chemical surface modifications

Surface modification of nanostructures is one approach to altering the properties of bioactive molecules. Because of this, the surface's wettability, chemical characteristics, and morphology vary in response, impacting the biocompatibility and cellular activity of the substrate surface.

Based on the proangiogenic action of peptides, gold nanostructures coated with functional peptides promote the formation of endothelial cell capillaries [224, 279, 285]. Adding oxygen- and nitrogen-containing functional groups to carbon nanotubes, such as amide and amine groups, can overturn the negative zeta potential of unfunctionalized carbon nanotubes. Using cation electrodeposition mediated by chitosan to coat nanostructures, it is possible to improve their interaction with the cell membrane that is negatively charged, resulting in increased activity, proliferation, adhesion, and survival of vascular smooth muscle cells and HUVECs [310]. In a study by Khor et al. they compared the characteristic features of polymeric nanomaterials functionalized by carboxylic acid/poly(ethylene glycol) methyl ether/methyl ester/tertiary amine ester with those of other modifications [120]. The findings suggest that NPs functionalized with tertiary amine ester have a more robust cell binding capacity in simulated and static intravascular fluids than other modifications.

On the other hand, the binding force between NPs modified with tertiary amine ester and cells is resistant to the impact of hemodynamic separation, even though larger size enhances the fluidic flow’s drag force. In addition, silica NPs modified by amine-terminated dendrimers demonstrate haematotoxicity, because their surfaces have positive charges that activate both plasminogen and fibrinogen at the same time [177]. Because the nanomaterials inside the framework may accidentally penetrate blood circulation or cells, it is essential to consider the safety and biological interactions of the nanomaterials. A nanostructured film consisting of poly (lactic–co-glycolic acid) that has been treated with sodium hydroxide is not favorable for endothelial cells' activities and functionality due to its surface's chemical effects [173]. It was achieved by employing a casting process that enhanced the endothelial cell’s density while removing the products of surface chemical reactions.

Immobilized growth factors in nanoscaffolds provide improved durability and biological activity. The angiopoietin 1 and VEGF in polylactic acid microspheres featuring a sustained release in a nano-based manner promotes the adipose MSC’s differentiation and proliferation into endothelial cells, which is beneficial for angiogenesis [91]. It has been established that a nanofibre scaffold packed with VEGF or FGF, when compared to a baseline nanofibre scaffold, dramatically inhibits thrombosis and promotes angiogenesis [83, 90]. The surface functionalization of nanofibre scaffolds with biomolecules (like heparin) results in increased angiogenesis at the implantation sites without external growth factors to be given to the patient [280, 288].

### Size

The literature review has thoroughly proven the relationship between the size of NPs and their effectiveness. According to Arvizo et al. [11], VEGF165's function is inhibited by gold nanostructures, and the inner diameter of gold nanomaterials is critical in this inhibition [11]. When comparing the diameter of 20 nm in gold and silica nanomaterials to that of the nanometer range, the former demonstrated an advantage in VEGF attachment throughout the biological medium and suppressed angiogenesis compared to the latter [110, 112]. The most efficient nanomaterials were those with a diameter of 20 nm [110, 112]. When compared to gold nanomaterials of dimensions of 5 nm and 10 nm, gold nanostructures of size 20 nm had the highest effect on the suppression of angiogenesis, mainly when they were administered at the same level as the other nanoparticle dimensions. That is because 95 percent of the protein bonded to the surface of that 20 nm was more effective than the 80 percent that was more effective on the surface of that 5 nm, resulting in the highest effect [11]. Strange results were reported by Guarneri et al. (2014), who revealed that silica nanostructures with a size of 25 mm showed no influence on the angiogenic responsiveness of endothelial cells even at the most significant concentrations (2.5 nM) [82]. While this was going on, additional research done by Jo et al. [111] discovered that bigger silicate nanostructures with an average diameter of 57 nm efficiently reduced retinal neovascularization induced by VEGF and inhibited ERK 1/2 activation through reduction of the phosphorylation of VEGFR-2 [111]. HUVECs were treated with nano-HAPs or spherical hydroxyapatite (HA) NPs having an 80 nm diameter, which reduced tube formation, cell migration, and NO generation more than HANPs with a 20 nm diameter [246]. With varied intensities of antiangiogenic impact, diverse physical and chemical features, like the sizes of microwave–radiofrequency (MW–RF) nanostructures and ultradispersed detonation diamond (UDD) nanomaterials, were demonstrated [81].

As a result of their research, they discovered that nanostructures from the selected papers have sizes ranging from 4 to 100 nm. The average diameter of 0.0 nm to 20 nm is the most often encountered. Nanomaterials with a diameter of 20 nm were more prevalent and were shown to be effective antiangiogenic agents.

### Shape

literature reviews have shown that silver nanomaterials with a spherical shape have an inhibitory impact on angiogenesis in various tissues [16, 309]. Nanostructures of spherical shape made of silica and gold influenced angiogenesis by inhibiting the process of blood vessel formation [110, 112]. The findings of Wierzbicki et al. [292] revealed that, among pristine carbon nanomaterials, multiwall nanotube microspheres (MWNTs) and pherical diamond NPs (ND) had the highest antiangiogenic characteristics and that sheet-like graphene NPs (GNs) and spherical graphite NPs (NG) had the most negligible effect. They showed varied results, although both had the same size and shape. On the other hand, spherical fullerene nanomaterials (C60) had the opposite effect, stimulating the growth of new blood vessels [292]. Researchers observed that the rod-shaped NP 80 was less effective in internalization into HUVECs than the HANPs having the diameter of NP 20, according to Shi et al. [246]. They have discovered that the particle shape and size significantly impact the absorption of nanomaterials by cells. They also asserted that the condition is a factor in determining the result of biological processes in addition to cellular absorption. As a result, researchers discovered that spherical nanomaterials are the most preferred form among various shapes in antiangiogenic investigations.

### The role and impact of ion release of NPs for vascularization

In addition to adapting the inherent features of biomaterials, such as their porous structure architecture, topography, bulk stiffness, and surface chemistry, one of the most important strategies for stimulating angiogenesis is the angiogenic molecules' controlled delivery through nanobiomaterials. Metallic ions have a substantial impact on angiogenesis as well as bone development. Some examples of metallic ions are copper, iron, strontium, magnesium, boron, titanium, zinc, silicate, and cobalt. Some of the ions, such as copper, zinc, and silver, give additional therapeutic benefits in addition to their pro-angiogenic properties, including antimicrobial properties and anti-inflammatory properties. A technique that does not include the use of growth factors to activate angiogenesis is one that involves the entrance as well as delivery of ions through nanobiomaterials. This can be accomplished in a number of different ways [118, 142]. Growth factors as well as metallic ions are two examples of representative angiogenic chemicals that can be incorporated into the composites [47].

In vitro, copper ions have the capacity to accelerate the healing of wounds in rats and stimulate the proliferation of endothelial cells in cultures [60]. It does this by upregulating the production of VEGF and promoting neovascularization. Copper ion has the ability to simulate hypoxia through its stabilization of the production of hypoxic inducing factor (HIF-1), which in turn stimulates VEGF genes as well as other genes [69, 92]. Hypoxia is a significant contributor to the induction of endothelial cells toward the creation of a functioning vasculature that supports blood supply. Furthermore, they showed that a tenfold increase in the copper dosage was required for the in vivo ingrowth of wound tissue, which was equal to 560 ng [22]. Therefore, it has been established that copper-loaded brushite scaffolds promote improved angiogenesis.

Through the production of circumstances that are analogous to hypoxia, it has been demonstrated that the cobalt ions' controlled release can stimulate angiogenesis in vivo and in vitro [43]. Furthermore, activation of the HIF-1 pathway by Co^2+^ ions can take place regardless of the total amount of oxygen present inside the cell [172]. The amount of HIF-1α that is present in the cytoplasm has a significant bearing on whether or not the HIF-1 pathway is activated. To be more specific, there are two conceivable outcomes. First, under conditions of normal oxygenation, HIF-1α is continually created, and subsequently it is destroyed by a mechanism involving the ubiquitin proteasome. Second, the hypoxic environment causes HIF-1α to become stable, which allows it to aggregate, go to the nucleus of the cell, and afterwards dimerize with HIF-1β to promote the expression of its targeted genes. Regardless of the amounts of oxygen in the environment, it is the responsibility of Co^2+^ ions to 'artificially' maintain the HIF-1α level by preventing the protein from degrading. As a consequence of this, a wide range of transcriptional responses take place, one of which is the overexpression of pro-angiogenic proteins (such as VEGF), which ultimately results in angiogenesis as well as overall improvements in oxygen levels [42]. According to the findings of one investigation, mesoporous bioglass scaffolds that contained less than 5% cobalt strongly provoked hypoxia, which led to an enhancement in VEGF protein production but also HIF-1 expression, in addition to an increase in the osteogenic gene expression [295]. Hoppe et al. created 1393 bioglasses that released cobalt ions and then conducted in vitro investigations using a fluid that was meant to resemble bodily fluid [95]. In a cobalteluting composite, both the VEGF gene expression and the alkaline phosphatase activity and protein production were significantly increased, which demonstrates the benefit of using a biomaterial that does not contain growth factors for vasculature [217]. In a separate investigation, HUVECs and hMSCs were employed to explore the in vitro angiogenic capabilities of beta-tricalcium phosphate that had been doped with cobalt. The findings provided an explanation for improved VEGF expression from hMSC along with improved network generation in HUVECs, indicating that cobalt ions may have a multifunctional role that facilitates the contacts between HUVECs and hMSC for the purpose of favorable angiogenesis. Recently, dual-ion delivery silicate microspheres doped with cobalt were created as drug-free multifunctional biomaterials [33]. These microspheres are capable of synergistically upregulating angiogenesis with the assistance of silicate ions triggering angiogenesis. Key angiogenic genes, such as the receptor kinase insert domain receptor (KDR), VEGF, and HIF1-alpha, were synergistically elevated in silicate-based microspheres that had been doped with 2.5 weight percent cobalt. Furthermore, neo-vascular development was greatly increased in the microspheres that carried dual ions, as demonstrated by their effects on a chicken chorioallantoic membrane model. According to research conducted by Gwang-BumIm et al. Fe ions delivered into hMSCs by bioreducible metal NPs keep improving cell migration, control ion-triggered intracellular ROS, as well as cell-homing effectiveness and increase angiogenic [102]. This is accomplished while simultaneously lowering cytotoxic effects. Endosome-triggered iron-ion-releasing NPs are particles that have been engineered to be sensitive to lower pH to make use of the low pH conditions (4–5) that are present in endosomes for in situ iron-ion release. As a result of the disparate redox potentials of iron and gold, only iron was able to be ionized and released from these unique NPs, but gold maintained its original state upon endocytosis of the NPs. A moderate rise in the rate of intracellular ROS was induced in hMSCs as a result of treating them with the optimum dose of NPs. This resulted in an increase in the production of HIF-1, which is an essential stimulant for the release of angiogenic growth factor from hMSCs. The treatment of hMSCs with NPs resulted in a considerable increase in gene expression and proteins linked to angiogenesis and lesion targeting. In a mouse model of wound healing, the transplantation of hMSCs by these NPs resulted in considerably improved angiogenesis. Using a galvanic replacement reaction of Cu NPs and Au precursor, Gwang-BumIm et al. conducted an innovative investigation, wherein they created Cu-based angiogenic metallic NPs [103]. These NPs were capable of stabilizing and upregulating intracellular activities in human adipose-derived stem cells (hADSCs). Their unique therapeutic Cu-NPs demonstrated significant benefits in cell therapy, including minimal mitochondrial dysfunction, decreased ATP consumption, reduced cytotoxicity, moderate ROS formation, plus improved angiogenic paracrine factor secretion, and better hypoxia inducible factor-1 alpha expression. Even in re-attached hADSCs, the expression of HIF-1 led to an increase in VEGF production that lasted for three days. Furthermore, these NPs increased other angiogenic factors as well as the percentage of copper that was exocytosed from human umbilical vein endothelial cells in vitro, which led to the robust growth of tubular formation via human umbilical vein endothelial cells.

According to the research that has been conducted so far, the form or embodiment, wherein highly purified silica or silicate nanoparticles are utilized appears to have a significant impact on the impact that they have on angiogenesis. Materials based on SiO_2_ that are biodegradable go through a process of gradual disintegration when they come into contact with biological fluids. This results in the release of silicate ions, which are significant on a physiological and biochemical basis. In co-cultured HUVECs and human dermal fibroblasts (HDFs), it was revealed that silicate ions provided from calcium silicate bioceramics played a crucial role in inducing angiogenesis. The concentration range for these ions was between 0.7 and 1.8 µg mL^−1^. To be more specific, the expression of VEGF was induced in HDFs by calcium silicate extracts, while the VEGF receptor 2 expression was elevated in HUVECs [144]. Both the generation of nitric oxide and endothelial nitric oxide synthase activation in these co-cultures were necessary steps in the beginning stages of angiogenesis. The same research team noticed a comparable pro-angiogenic mechanism when human aortic endothelial cells were cultivated with dissolving extracts from akermanite, which included Mg^2+^, Ca^2+^, and silicate ions [322]. In addition, akermanite stimulated the growth of new blood vessels, while it was inserted in a rabbit femoral condyle prototype for a period of 2 and 4 months in vivo, respectively. It is possible for high dosages of silicate substances to trigger cytotoxic effects because of the greater concentrations of ionic dissolving byproducts. This may further enhance the pH of the cultured cells, causing excessive alkalinity that makes it difficult for cells to survive [119]. It is also important to highlight that, although silicate ions can in fact evoke proangiogenic effects, the integration of metallic dopants with more effective angiogenic impacts into SiO_2_ bioglass mixtures, particularly mesoporous silica nanostructures, may be a superior strategic approach to encourage vascular sprouting. This is because mesoporous silica nanostructures have a higher concentration of silicate ions [294].

Studies conducted on rats in vivo that utilized calcium silicate bioceramics doped with strontium provided conclusive evidence of the function that strontium ions play in the process of angiogenesis [297, 331, 332]. The release of strontium ions led to an enhancement in both the angiogenic gene VEGF expression as well as endothelial cell proliferation. It is important to highlight that the effectiveness of strontium–calcium silicate was shown in osteoporotic significant calvarial defects that had a great angiogenic blood supply [150]. Strontium ions may also indirectly stimulate angiogenesis by inducing macrophages to release pro-angiogenic factors. According to the findings of the study, strontium-containing bioglass microspheres were capable of polarizing macrophages from the M1 phase to the M2 phase. Furthermore, the M2 phenotype was demonstrated to produce PDGF-BB, which supported neo-angiogenesis both in vivo and in vitro [329].

Previous research found that a higher level of magnesium ions, specifically 10 mM, was able to modify the activities of endothelial cells in vitro [167]. These activities included angiogenic factor secretion, endothelial proliferation, and nitric oxide generation. Magnesium is also essential to the functional and structural integrity of endothelial cells, which helps to inhibit atherosclerosis as well as maintain vascular homeostasis. Magnesium ions, just like strontium ions, have been found to affect the phenotype of macrophages and, as a result, indirectly stimulate angiogenesis even during the process of bone repair [190].

In addition to this, zinc is capable of exerting regulation on the phenotype of the macrophage that is essential for angiogenesis [252]. Zinc, which is present in zinc oxide substances, is thought to be a major ROS producer that is essential for triggering angiogenesis mediated by VEGF signaling [89]. For instance, upregulation of proangiogenic genes such as VEGF and FGF may occur if zinc oxide nanomaterials are included in electrospun PCL scaffolds [185]. In another instance, researchers investigated the signaling route involved in angiogenesis mediated by ZnO [26]. It was discovered that ZnO's creation of ROS stimulated a pathway including MAPK, eNOS, and Akt, which then resulted in cGMP-dependent angiogenesis and nitric oxide synthesis. Given that zinc is an element that is necessary for the proper functioning of osteoblasts, it is reasonable to assume that ZnO, as well as other varieties of zinc, can serve as an effective ion for the process of angiogenesis [242].

In one experiment using bioglass based on silica, titanium dioxide was used in place of silicon in one experiment. This replacement has the potential to cause superior angiogenesis as well as the ion Ti, which has been suggested as a potential effective angiogenic promoter [5].

Cerium is another element that is receiving a lot of attention because of its capacity to stimulate angiogenesis by modifying the intracellular oxygen environment. This, in turn, protects progenitor or endothelial cells from the effects of ROS stress and speeds up angiogenesis by activating HIF-1 [165, 300]. An interesting study that was conducted by Das and colleagues associated cerium oxide NPs with angiogenesis both in vivo and in vitro [56]. They demonstrated that physical and chemical properties, such as the ratio of Ce^3+^ to Ce^4+^ on the surface, shape, size, and surface charge, all played a part in regulating the process of angiogenesis. For instance, the catalytic behavior of cerium oxide NPs increased with a greater surface area related to ratio of Ce^3+^/Ce^4+^, which finally regulated oxygen level in intracellular medum and angiogenesis. However, researchers were unable to determine the direct role that ions released from nanomaterials play in the process of angiogenesis.

## Conclusions

The process of angiogenesis is kept in a state of natural equilibrium by a number of factors that are both pro- and anti-angiogenic; nevertheless, an imbalance between these factors can result in abnormal angiogenesis, which is strongly linked to a variety of conditions. Angiogenesis and vascularization are crucial physiological processes that play a significant part in various functions, such as the female menstrual cycle, wound healing, embryonic growth and development. On the other hand, it is engaged in the pathophysiology of various pathological illnesses, such as cancer, rheumatoid arthritis, cardiovascular disease, psoriasis, diabetic nephropathy, and other diseases of the circulatory system. Traditional angiogenesis treatments based on growth factors and cells are restricted in their ability to be effective for different reasons. The hurdles that must be overcome to resolve these concerns include durability, cytotoxicity, manufacturing cost, better dissolution rate, and biocompatibility. Nanotechnology and nanomedicine have emerged as effective options for traditional ways of addressing the challenges of these technologies. Numerous nanostructures have been devised and produced by different research organizations worldwide for therapeutic angiogenesis, and they have shown great promise both in vivo and in vitro. The majority of the successful medication-mediated anti-angiogenic treatments are restricted due to the unavoidable development of drug resistance. Since the beginning of this decade, nanomedicine has been used to solve a wide variety of diseases in the fields of medicine and biology. When it comes to cancer treatment, anti-angiogenic nanomaterials are anticipated to bring about a sea change in the medical landscape over the next decade. Depending on the level of development and the improvements that have been made, anti-angiogenic nanostructures can either be administered on their own or perhaps in conjunction with other anti-cancer medicines, peptides, or siRNA. The optimizing of dosage and duration of the NPs also has to be investigated, and it is imperative that special attention be paid to ensure the safety of antiangiogenic therapy across a broad spectrum of diseases. Before using these novel anti-angiogenic NPs in clinical investigation, various factors, such as metabolic long-term fate (in vivo and in vitro), biosafety, effectiveness, interaction of the particulates with immune cells, possibility of long-term toxic effects, and pharmacodynamic and pharmacokinetic survey in experimental animals, should be carefully investigated. The nanomedicine method provides researchers with the opportunity to generate unique nano-engineered anti-angiogenic NPs, which in the near future may prove to be the most promising and practically viable alternative technology for the treatment of cancer. The use of angiogenesis blockers in conjunction with a technique based on nanomedicine could prove to be a beneficial and potentially effective new therapy approach for cancer research.

As a means of overcoming the limitations of traditional methods, nanomedicine and nanotechnology have emerged as good alternatives to traditional methods. For the purpose of therapeutic angiogenesis, a number of different research organizations from all around the world have created and manufactured metal NPs. These NPs have shown outstanding promise at both the in vivo and in vitro levels. Nevertheless, to successfully translate these findings into clinical practice, there are a number of obstacles that must be addressed. The growing use of NPs in therapeutic angiogenesis has brought about the requirement for the development of more advanced production and characterization methods. In addition to this, the development of innovative methods for the production of NPs as well as technologically sophisticated characterization instruments is essential. For use in biological applications, it is desirable to have NPs made of metal that are durable, have the same size and shape (monodispersed), and have desirable absorption coefficient, magnetic, fluorescence, and electric characteristics. After the NPs have been created, a key difficulty that has to be tackled is forming them so that they can be delivered to the appropriate location of action. It is also vital to do research on the biocompatibility, toxic effects, and bioretention of these NPs to ascertain their long-term destiny and the possible risks associated with them. In addition, developing realistic models of angiogenesis ex vivo, in vitro, and in vivo is essential to properly screen a collection of nanostructures for therapeutic angiogenesis. Furthermore, to optimize the creation of improved nanocandidates for the purposes of achieving augmented therapeutic potential, it is extremely important to have a comprehensive understanding of the comprehensive molecular mechanisms as well as signaling cascades that underlie the pro-angiogenesis that is stimulated by the NPs. In addition, the capability of NPs to transport medications and biomolecules to the location, where they will be most effective has been widely established. Therefore, the pro-angiogenic metallic NPs can be created for the delivery of various pro-angiogenic medications or growth factors for the purpose of generating an improved angiogenesis effect in diseases that develop as a result of insufficient blood vasculature. To use pro-angiogenic NPs in clinical settings, it is necessary to have a comprehensive understanding of important criteria, including therapeutic window, biological half-life, dose and route of administration, and pharmacokinetic parameters like biodistribution, absorption, metabolism, and removal of the NPs. We have a strong belief that NPs might emerge as effective options for the treatment of angiogenesis-related diseases. This belief is based on the significance of NPs, which is proven by the number of NPs in clinical and preclinical studies.

## Data Availability

Not applicable.
